# Obesogenic diet in mice compromises maternal metabolic physiology and lactation ability leading to reductions in neonatal viability

**DOI:** 10.1111/apha.13861

**Published:** 2022-08-03

**Authors:** Samantha C. Lean, Alejandro A. Candia, Edina Gulacsi, Giselle C. L. Lee, Amanda N. Sferruzzi‐Perri

**Affiliations:** ^1^ Centre for Trophoblast Research, Department of Physiology, Development and Neuroscience University of Cambridge Cambridge UK; ^2^ Department for the Woman and Newborn Health Promotion Universidad de Chile Santiago Chile

**Keywords:** adiposity, diet, fetus, lactation, metabolism, obesity, pregnancy

## Abstract

**Aims:**

Diets containing high‐fat and high sugar (HFHS) lead to overweight/obesity. Overweight/obesity increases the risk of infertility, and of the pregnant mother and her child for developing metabolic conditions. Overweight/obesity has been recreated in mice, but most studies focus on the effects of chronic, long‐term HFHS diet exposure. Here, we exposed mice to HFHS from 3 weeks prior to pregnancy with the aim of determining impacts on fertility, and gestational and neonatal outcomes.

**Methods:**

Time‐domain NMR scanning was used to assess adiposity, glucose, and insulin tolerance tests were employed to examine metabolic physiology, and morphological and proteomic analyses conducted to assess structure and nutrient levels of maternal organs and placenta.

**Results:**

Fertility measures of HFHS dams were largely the same as controls. HFHS dams had increased adiposity pre‐pregnancy, however, exhibited exacerbated lipolysis/hyper‐mobilization of adipose stores in late pregnancy. While there were no differences in glucose or insulin tolerance, HFHS dams were hyperglycemic and hyperinsulinemic in pregnancy. HFHS dams had fatty livers and altered pancreatic islet morphology. Although fetuses were hyperglycemic and hyperinsulinemic, there was no change in fetal growth in HFHS dams. There were also reductions in placenta formation. Moreover, there was increased offspring loss during lactation, which was related to aberrant mammary gland development and milk protein composition in HFHS dams.

**Conclusions:**

These findings are relevant given current dietary habits and the development of maternal and offspring alterations in the absence of an increase in maternal weight and adiposity during pregnancy, which are the current clinical markers to determine risk across gestation.

## 1 INTRODUCTION

A “Western style” diet high in fat and sugar is contributing to a pandemic of raised body mass index (BMI) and obesity in developed countries.^1^, ^2^, ^3^, ^4^ As a result, the majority (52.7%) of women are overweight (BMI >25 kg/m^2^) or obese (BMI >30 kg/m^2^) at the point of conception^5^, ^6^, ^7^ leading to problems in both achieving,^8^ and maintaining a healthy pregnancy. For example, increased BMI is associated with an increased risk of pregnancy loss and compromised fetal development and wellbeing.^9^, ^10^ It is also linked to a greater risk of the infant developing health problems during childhood,^11^ as well as hypertension, heart disease, diabetes, and obesity in adult life.^12^, ^13^ As a result, obstetric health care costs are 23% and 37% higher for overweight and obese mothers compared to healthy weight mothers, respectively.^14^ Moreover, when obese women also struggle to conceive, the cost of each livebirth is 70% higher for ovulatory and 100% higher for anovulatory obese compared to healthy weight women who struggle to conceive.^15^ Combined with the programming effects of obesity on offspring disease risk later in life, this renders maternal obesity not just an increasing strain on national health services, but a global health threat.^16^

The impact of high maternal BMI on fertility and pregnancy outcomes may be due to a number of reasons. Women who are overweight/obese can have menstrual cycle disturbances with anovulatory cycles.^17^ Furthermore, there is reduced oocyte quality^18^, ^19^ and decreased chances of implantation^8^ due to endometrial disturbances in decidualization and gene expression.^20^, ^21^, ^22^ This leads to a greater proportion of women with raised BMI relying on assisted reproductive technology services. Within fertility treatment, 10% fewer obese anovulatory women will achieve pregnancy through fertility treatment than healthy weight anovulatory women.^15^ If pregnancy is achieved, early pregnancy loss (<20 weeks) is significantly higher (OR 1.67) in overweight versus healthy weight women^23^, ^24^ regardless of conception method.

Raised BMI also increases the risk of a number of maternal pregnancy complications, such as gestational hyperglycemia, gestational diabetes mellitus (GDM), and non‐alcoholic fatty liver disease.^25^, ^26^, ^27^, ^28^ In part, these poor maternal outcomes may reflect aberrant maternal metabolic physiology in pregnancy. Normal pregnancy involves the development of hyperinsulinemia, insulin resistance, hyperglycemia, and hyperlipidemia in the mother to allow for nutrient partitioning to the fetus for growth and development.^27^, ^28^, ^29^ Metabolic adaptations in pregnancy require careful orchestration of changes in insulin sensitivity by maternal metabolic organs, including adipose and liver, as well as alterations in insulin production by the pancreas. When the mother is obese, these adaptations in metabolic physiology may be mis‐appropriately induced or become exaggerated, and likely contribute to the development of gestational diabetes and fetal growth disturbances, namely large for gestational age (LGA) infants.^30^ However, in a subset of obese women, infants may also be born normal weight or can instead be small for gestational age (SGA) or show fetal growth restriction (FGR).^31^, ^32^, ^33^ Moreover, there are data to suggest that obesity leads to impaired breastfeeding^34^ which could impact neonatal development and growth.^35^

A “Western style” high‐fat, high sugar (HFHS) diet has been recreated in mouse models replicating weight gain, maternal metabolic maladaptations in pregnancy, abnormal fetal growth, and offspring adult health programming.^31^ However, the majority of studies have focussed on long‐term exposure to an obesogenic diet (from anywhere between 4 weeks to 14 weeks prior to pregnancy) with established maternal obesity as characterized by significant increases in body weight.^36^, ^37^ By contrast, the effects of shorter‐term consumption of such diets on fertility, pregnancy and offspring outcomes, and contribution of changes in key maternal organs to these outcomes, are far less explored.^38^ In particular, whether there may be changes in maternal hepatic nutrient handling, pancreas insulin production, or mammary lobuloalveolar development, as these could impact metabolic health in pregnancy and the provision of resources for fetal and neonatal growth and survival. This is important for understanding the impact of mothers changing their dietary habits just before and during pregnancy and who may not show overt changes in body weight, rather than chronically obese mothers who may also have other underlying problems, like insulin resistance.

This study aimed to determine the effect of a HFHS diet from 3‐weeks prior to pregnancy and known maternal adiposity on fertility, gestational, and neonatal outcomes in mice. In particular, we examined the ability to achieve and maintain pregnancy, placental structure, fetal growth and maternal metabolic physiology, lactational indices and neonatal survival in mice fed a HFHS diet compared to controls.

## 2 RESULTS

### 2.1 Greater increase in maternal adiposity with HFHS diet pre‐pregnancy

At 6 weeks of age, female C57Bl/6J mice were randomized to remain on the control (RM3) chow or provided with a customized high‐fat high sugar (HFHS) diet (*n* = 20/group) for 3 weeks prior to, and during, pregnancy (study design; Figure S1). As determined by weight gain monitoring and time‐domain nuclear magnetic resonance (TD‐NMR) scanning, no differences were seen in initial (pre‐diet allocation) maternal body weight, adiposity, and lean mass between chow and HFHS fed mice (Table 1). There was also no effect of a HFHS diet on pre‐pregnancy body weight and change (delta) in body weight since commencing the diet 3 weeks earlier. However, HFHS mice exhibited a greater increase in adiposity, but lower gain of lean mass over those 3 weeks pre‐pregnancy, when compared to chow‐fed counterparts (Table 1, *p* < 0.001).

**TABLE 1:** Maternal body composition and fertility parameters

Body composition	Control (*n* = 40)	HFHS (*n* = 41)	*p* Value
Body weight			
Initial	17.81 ± 0.33	17.71 ± 0.29	NS
Pre‐pregnancy	21.58 ± 0.39	22.22 ± 0.46	NS
Delta	3.76 ± 0.28	4.52 ± 0.43	NS
Adiposity (g)			
Initial	2.018 ± 0.08	2.14 ± 0.08	NS
Pre‐pregnancy	2.067 ± 0.08	4.95 ± 0.29	**<0.0001**
Delta	0.07 ± 0.08	2.77 ± 0.27	**<0.0001**
Adiposity (%)			
Initial	11.22 ± 0.43	12.31 ± 0.43	NS
Pre‐pregnancy	9.59 ± 0.32	21.86 ± 0.95	**<0.0001**
Delta	−0.77 ± 0.59	9.61 ± 0.95	**<0.0001**
Lean (g)			
Initial	13.12 ± 0.33	12.64 ± 0.26	NS
Pre‐pregnancy	15.82 ± 0.29	13.84 ± 0.18	**<0.0001**
Delta	2.68 ± 0.21	1.18 ± 0.25	**<0.0001**
Lean (%)			
Initial	72.53 ± 0.73	71.27 ± 0.68	NS
Pre‐pregnancy	73.33 ± 0.26	62.90 ± 1.00	**<0.0001**
Delta	0.84 ± 0.70	−8.32 ± 1.06	**<0.0001**
*Fertility measure*			
Pregnancy success rate			
# plugs	41	36	
# plugs resulting in pregnancy	36	33	
%All mating attempts	56.3%	52.6%	NS
%All plugs	87.1%	87.7%	NS
Days to plug (mean ± SEM)	2.86 ± 0.28	3.06 ± 0.24	NS
Plug on 1st night (*n*[%])	12 (30.0%)	7 (17.5%)	NS
Missed plug			
# NPFs	24	20	
# Missed plug‐pregnancy	2	4	
%All mating attempts	3.08%	7.14%	NS
%All NPFs	8.33%	20.0%	NS
# mating attempts‐pregnancy (mean ± SEM)	1.78 ± 0.14	1.38 ± 0.11	**0.0398**
Rate of infertility *n* (%)	3 (7.5%)	2 (4.8%)	NS

*Note* : Initial = at point of diet allocation (6 weeks of age); Pre‐pregnancy = ~3 weeks post‐diet allocation and immediately prior to mating; plug = evidence of copulation plug; NPFs = no plugs found during mating period; missed plug ‐ pregnancy = no plug found during mating period but resulted in pregnancy; %All mating attempts = as a proportion of all mating periods with males regardless of if plug was found or pregnancy resulted; %All plugs = as a proportion of all matings that resulted in a plug regardless of whether it resulted in a pregnancy; Day to plug – number of days paired with male until a plug was found that resulted in a pregnancy; #mating attempts – pregnancy = number of mating periods with male required before a pregnancy was achieved, infertility = either three plugs found that did not result in pregnancy, or five mating attempts that did not result in pregnancy regardless of whether plugs were found. Data analyzed by Student's *t*‐test or Fisher's exact for continuous and categorical data, respectively; significance at *p* < 0.05. The bold values are to indicate the significant *p* values.

Abbreviation: NS, not significant.

### 2.2 Increased maternal adiposity due to a HFHS diet does not have a notable impact on fertility

Markers of fertility were measured based on mating attempts and rates of pregnancy. Rates of infertility (inability to conceive), days to plug, and plug to pregnancy rate were not different between control and HFHS diet‐fed mice (Table 1). The number of HFHS mice that plugged on the first night of being paired with a male was almost half of those fed a control diet (17.5% vs 30.0%) although this did not reach statistical significance (*p* = 0.1487). Despite this, there was a small, but significant reduction in the number of mating attempts required to achieve a pregnancy in the mice fed the HFHS diet (*p* < 0.05).

### 2.3 HFHS diet‐fed dams show a hyper‐mobilization of adiposity across pregnancy

**FIGURE 1:**
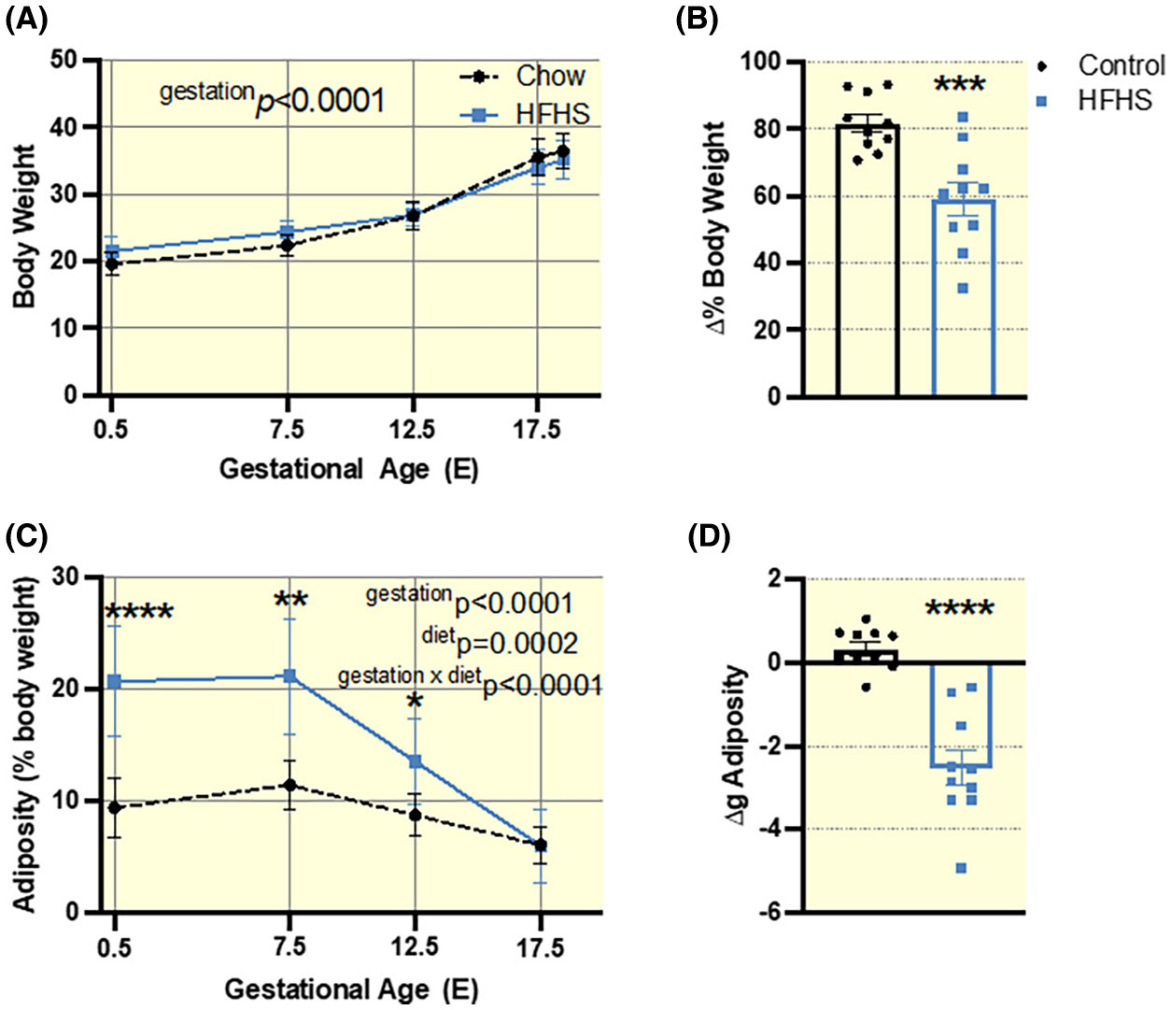
Maternal bodyweight and adiposity during pregnancy in dams fed a HFHS or control diet (*n* = 10/diet). (A). Body weight (g) at E0.5, 7.5, 12.5 17.5 and 18.5. (B). Change in body weight (%) from E0.5 to E17.5 from E0.5. (C) Adiposity as a percentage of body weight at E0.5, 7.5, 12.5 and 17.5. (D) Change in adiposity (∆g) between E0.5 and E17.5. Data presented as individual values and/or mean ± SEM, analyzed by Two‐way ANOVA repeated measures with multiple comparisons (A and C) or student *t*‐test (B and D); *p < 0.05, **p < 0.01, ****p < 0.0001.

Although both control and HFHS mice gained weight across gestation (Figure 1A), those fed a HFHS diet gained significantly less weight between embryonic days (E) 0.5 and 17.5 (Figure 1B). This difference may, in part, be due to differences in maternal adipose deposition in pregnancy. Mice fed a control diet, on average, gained ~10.5% adiposity by E7.5 (Figure 1C) and, although they showed a lower percentage of adiposity by E17.5 (6.12%) due to increasing gravid body weight, on average had a net gain in adiposity across pregnancy (between E0.5 and 17.5 Figure 1D). Despite starting with higher adiposity levels, mice fed the HFHS diet exhibited a drastic reduction in adiposity as a percentage of body weight (20.7% at E0.5 to 6.0% at E17.5; Figure 1C), and exhibited a net loss of percentage adiposity between E0.5 and 17.5 (Figure 1D). This may indicate a hyper‐mobilization of adipose depots in HFHS‐fed mice between E7.5 and 17.5 of pregnancy.

### 2.4 Maternal insulin and glucose handling are not overtly altered, but circulating lipids are decreased in HFHS diet ‐fed pregnant dams

**FIGURE 2:**
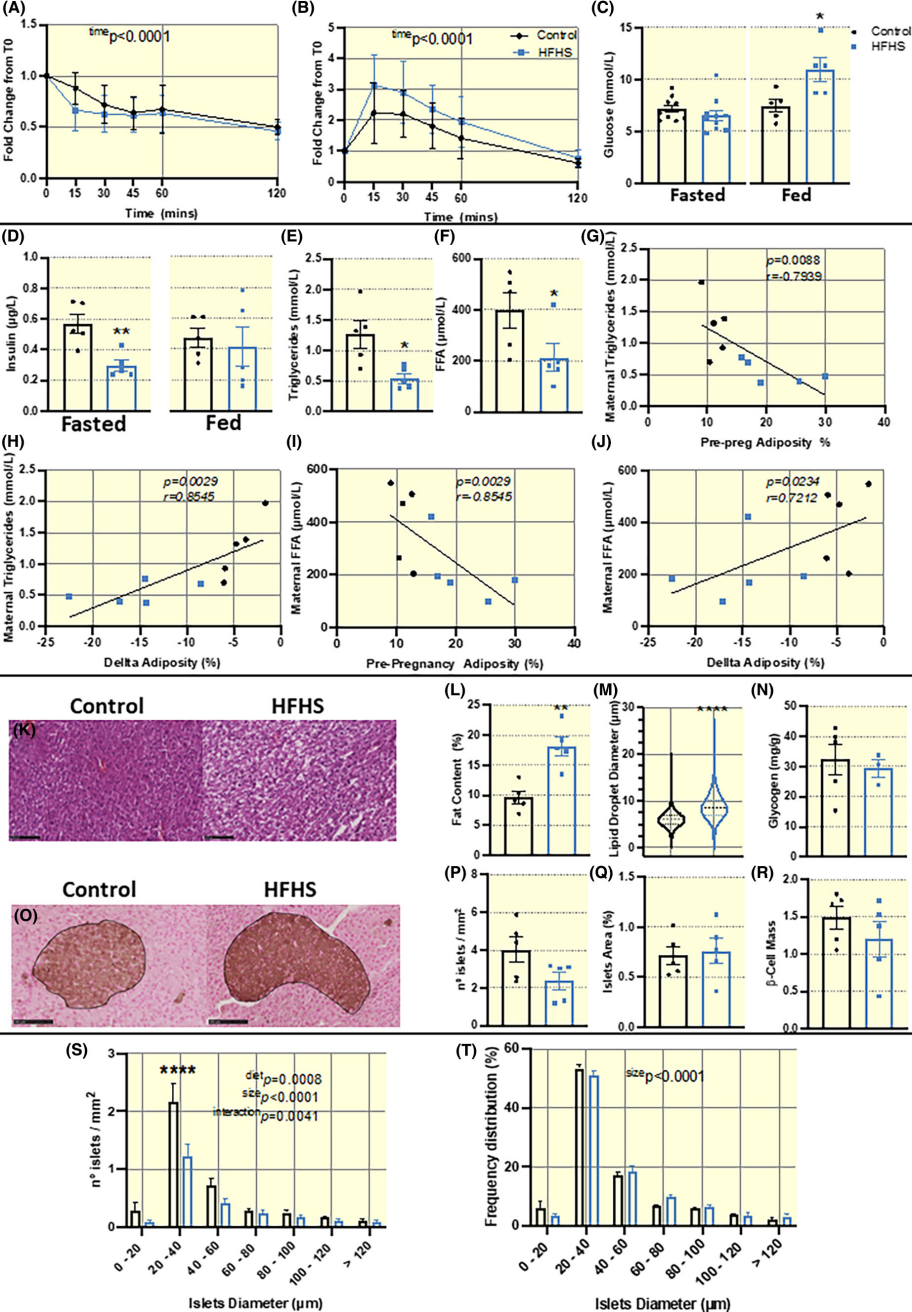
Maternal metabolic physiology assessed at E17.5 or E18.5 in dams fed a HFHS or control diet. Fold change in blood glucose response to (A) insulin and (B) glucose over 120 min at E17.5 analyzed by Two‐way ANOVA with repeated measures and multiple comparisons. (C) Fasted (4 h, E17.5) and fed (E18.5) blood glucose. (D) Insulin levels in fasted (4 h) and stimulated states (0′ and 15′ post bolus glucose injection) at E17.5 (*n* = 10/group) and fed state at E18.5 (*n* = 5/group). (E and F) Circulating maternal triglycerides and non‐esterified free fatty acids (FFA) at E18.5. (G–J) Correlations of circulating triglycerides and FFA to pre‐pregnancy %adiposity and ∆%adiposity across gestation (*n* = 5/group, groups combined), analyzed by Spearman‐Rank test. (K) Representative 20x magnification images of H&E stained liver sections (scale bar = 100 μm). (L) Hepatic fat content (% of wet tissue weight) and (M) assessment of liver lipid droplet (steatosis) diameter represented by violin plot and histogram of relative distribution (%). (N) Hepatic glycogen content (mg/g tissue). (O) Representative 20x images of pancreas sections stained by immunohistochemistry for insulin (scale bar = 100 μm). (P) Number of islets per/mm^2^ of tissue, (Q) islet area (%) and (R) β‐cell mass (mg), as well as (S and T) frequency distribution in number and % or islet diameter analyzed by 2‐way ANOVA (diet and size frequency) with multiple comparisons. Histological and biochemical analysis of maternal organs in K‐T assessed at E18.5 in pregnancies fed a HFHS or control diet (*n* = 5/group). Data presented as individual values and/or mean ± SEM and analyzed by student *t*‐test unless otherwise stated; *p < 0.05, **p < 0.01, ****p < 0.0001.

As indicated by an insulin tolerance test, dams fed the HFHS diet did not exhibit alterations in maternal insulin sensitivity on E17.5 (Figure 2A and area above the curve [AAC] mean ± SEM: 38.63 ± 6.83 vs 46.34 ± 7.22 mmol/L/min in control vs HFHS, respectively, *p* = 0.6905). Similarly, as informed by a glucose tolerance test, maternal glucose handling on E17.5 was not significantly different between HFHS and control dams (Figure 2B and area under the curve [AUC] mean ± SEM: 61.15 ± 24.95 vs 112.9 ± 33.95 mmol/L/min in control vs HFHS, respectively, *p* = 0.1508). Maternal blood glucose concentrations were significantly higher in the fed, but not fasted state (fasted for 4 h) in the HFHS compared to the control pregnant dams (*p* = 0.0239, Figure 2C). Circulating insulin was lower in fasted HFHS mice versus controls (*p* = 0.0051, Figure 2D), but this was not the case for insulin concentrations in the fed state. Both circulating triglycerides and free fatty acids were significantly lower in HFHS diet‐fed mice compared to controls at E18.5 (fed state, *p* = 0.0159 and 0.0317, respectively, Figure 2E,F). Both circulating triglycerides and free fatty acids showed strong negative correlations to pre‐pregnancy percentage adiposity and positive correlations to the change in percentage adiposity between pre‐ and late pregnancy (Figure 2G–J).

### 2.5 Maternal organ weights and hepatic and pancreatic morphology are altered in HFHS diet fed pregnant dams

At E18.5, maternal whole and hysterectomised body weight were not altered by a HFHS diet (Table S1). When expressed as a proportion of hysterectomised body weight, mice fed a HFHS diet had greater retroperitoneal adipose depot mass compared to control diet‐fed mice, although renal and gonadal fat depots were not different between dietary groups (Table S1). In mice fed the HFHS diet, the weight of multiple maternal organs was increased; brain, heart, liver, and mammary gland (all *p* < 0.05, except for a statistical tendency for mammary gland; *p* = 0.06), and pancreatic weight decreased when compared to controls (Table S1).

Further investigations revealed that the increase in liver weight in HFHS diet‐fed dams is related to an increase in liver fat content (10% vs 19% fat content, *p* = 0.0079, Figure 2K,L). Histological analysis of the maternal liver, showed an increase in fat droplet diameter (*p* < 0.0001, Figure 2M), indicating a degree of steatosis in the liver of mice fed a HFHS diet. As determined by an enzymatic assay, liver glycogen content was however not different between dietary groups (Figure 2N). In addition, histological investigation of the maternal pancreas revealed that, although number and area of islets and β‐cell mass were not altered (Figure 2O–R), there was a reduction in the number of small islets with a diameter of 20–40 μm in dams fed the HFHS diet (*p* < 0.0001, Figure 2S). Islets of that size (20–40 μm) are the most abundant in both control and HFHS diet‐fed pregnant mice (Figure 2T).

### 2.6 Fetal weight is not affected, even though fetal glucose and insulin concentrations are elevated in dams fed a HFHS diet

**FIGURE 3:**
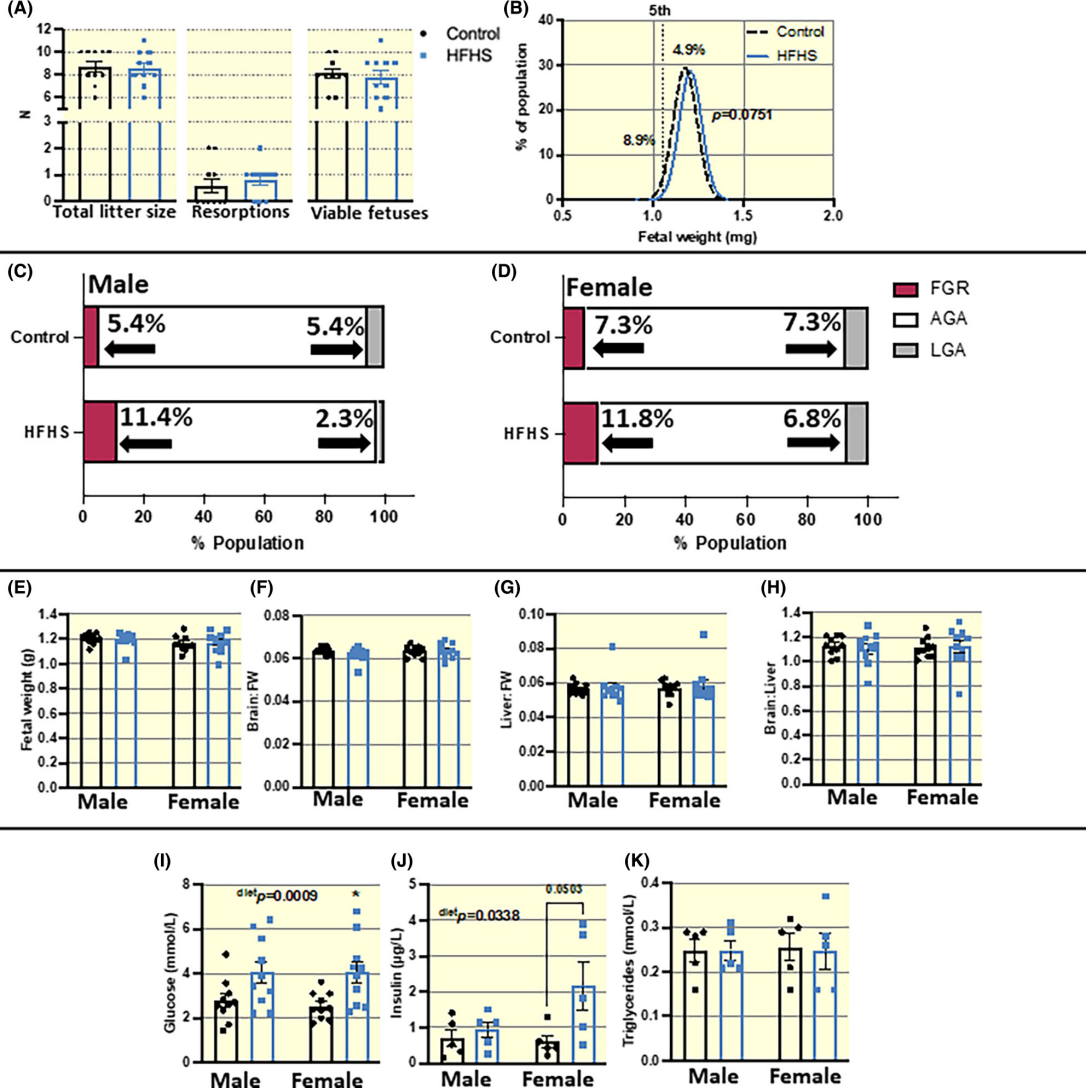
Fetal outcomes assessed at E18.5 in dams fed a HFHS or control diet. (A) Total litter size (resorptions + viable fetuses), resorptions and viable fetuses per litter (*n* = 10/diet). (B) Fetal weight distribution with 5th centile marker of control population with no consideration of fetal sex. (C and D) Classification of fetuses within the litter as exhibiting small for gestational age (SGA, <5th centile), appropriate for gestational age (AGA, 5th‐95th centile) and large for gestational age (LGA, >95th centile) of control populations for each sex. For C (males), the cut‐off for SGA was 1.099 g and for D (females), it was 1.030 g. There were no statistically significant differences in the proportion of male and female fetuses classified as SGA or LGA between control and HFHS groups, as determined by Fisher's Exact test. (E) Fetal weight (FW), (F) brain:fetal weight, (G) liver:fetal weight and (H) brain:liver weight ratios as markers of growth symmetry (*n* = 10/group). (I–K) Concentrations of circulating fetal glucose, insulin and triglycerides (*n* = 5 from different litters/group). E–H represents single value of the closest to average pup per sex per litter. Data presented as individual values and/or mean ± SEM and analyzed by Two‐way ANOVA (diet and sex) with multiple comparisons unless stated otherwise, *p < 0.05.

No differences were seen in the number of total conceptuses (resorptions + viable fetuses), resorptions, or viable/live fetuses between control and HFHS diet‐fed dams at E18.5 (Figure 3A). Moreover, there was no overall significant change in fetal weight distribution or average fetal weight between control and HFHS diet‐fed mice (Figure 3B,E). When data were split by fetal sex, the proportion of fetuses that fall below the 5th centile of the control population (hence, classified as small for gestational age; SGA) doubled from 5.4% to 11.4% in males (Figure 3C), and increased by 62% (from 7.3% to 11.8%) in female fetuses of HFHS diet‐fed mice (Figure 3D). Moreover, the proportion of males falling above the 95th centile of the control group (hence, LGA) was halved in mice fed the HFHS diet (Figure 3C). However, the frequencies of SGA or LGA for each fetal sex were not significantly different between control and HFHS diet‐fed dams. There was also no evidence of asymmetric fetal growth in HFHS dams, as indicated by the ratio of the fetal brain and liver to body weight, and the ratio of brain to liver weight in either sex (Figure 3F–H). However, in dams fed a HFHS diet, fetal blood glucose (Figure 3I) and insulin (Figure 3J) concentrations were increased in both sexes compared to controls; with pairwise comparisons revealing that the effect that was stronger for females. Fetal triglyceride concentrations were not affected in either sex by a maternal HFHS diet (Figure 3K).

### 2.7 Placental morphology is altered in dams fed a HFHS dietconcentrations are elevated in dams

**FIGURE 4:**
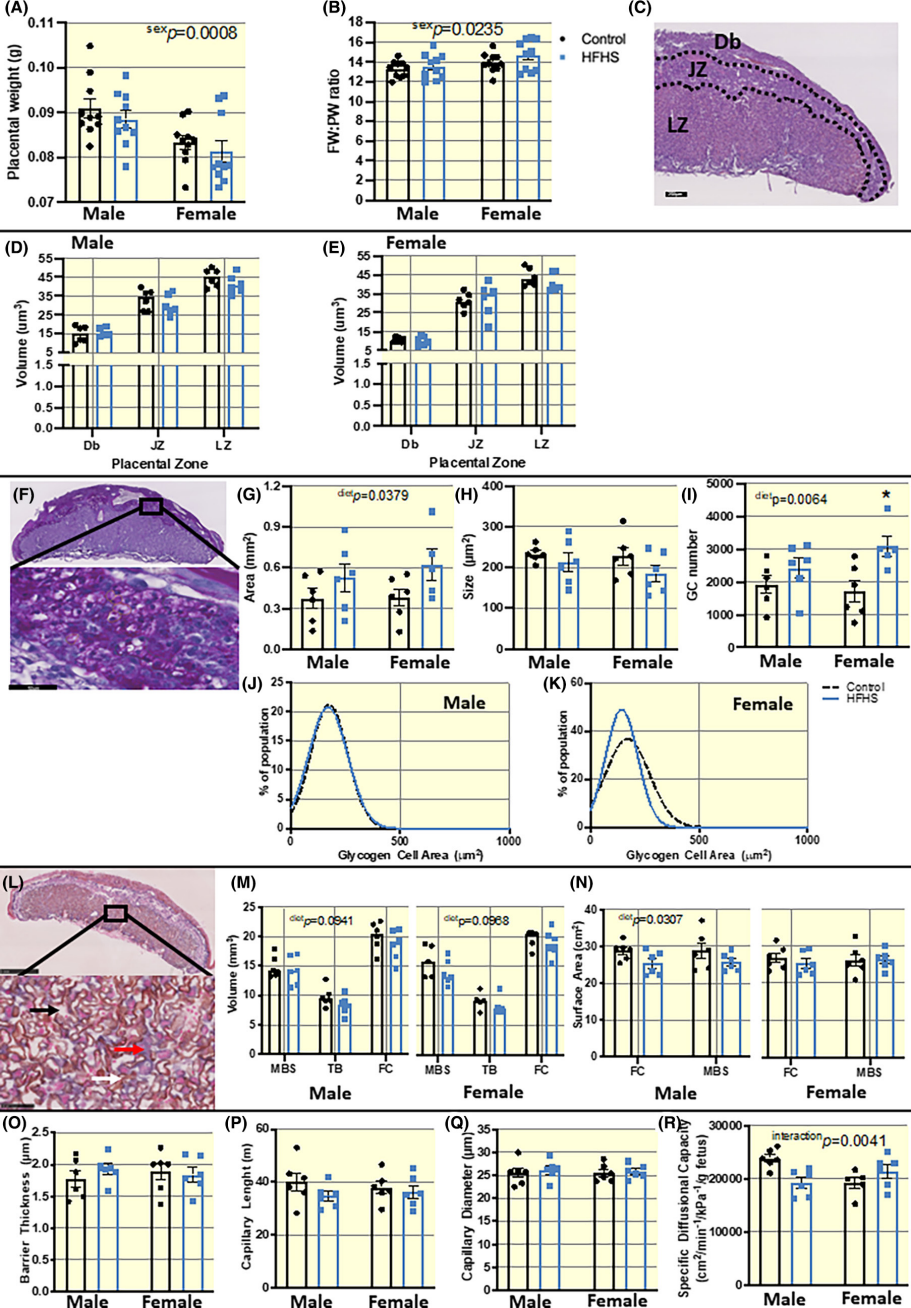
Placental structure assessed at E18.5 in dams fed a HFHS or control diet. (A) Placental weight and (B) fetal weight:placental weight (FW:PW ratio) with *n* = 10/group and data represents single value of the closest to average pup per sex per litter. (C) Representative image of H&E stained placenta to show different zones; decidua basalis (Db), junctional zone (JZ) and labyrinth zone (LZ) annotated (scale bar = 250 μm). (D and E) Placental compartment volumes per fetal sex. (F) Representative images PAS stained section (scale bar = 50 μm) and (G) estimated glycogen cell stained area, (H) glycogen cell size, (I) glycogen cell number, and (J and K) glycogen cell size frequency distribution. Data from *n* = 6 from different litters/group. (L) Representative image of cytokeratin and lectin dual immunohistochemistry at low and high magnification (scale bar = 1 mm and 50 μm, respectively) with maternal blood space (MBS, black arrow), trophoblast (TB, red arrow) and fetal capillaries (FC, white arrow) annotated. (M) Volume of LZ constituents and (N) surface area of FC and MBS. (O) Barrier thickness, (P and Q) FC diameter and length and (R) specific diffusing capacity. Data from *n* = 6 from different litters/group. Data presented as individual values and/or mean ± SEM and analyzed by Two‐way ANOVA (diet and sex). *p < 0.05.

To provide information on the mechanisms contributing to the lack of alterations in fetal growth despite fetal hyperglycemia and hyperinsulinemia in HFHS dams, placental size, and morphology were determined. A maternal HFHS diet did not alter placental weight or placental efficiency (fetal to placental weight ratio), although overall, females had slightly smaller and more efficient placentas than males at E18.5 (Figure 4A,B). As informed by exhaustive placental sectioning, hematoxylin and eosin staining and unbiased stereological analysis, a maternal HFHS diet also did not affect the volume of the maternal decidua basalis (Db), endocrine junctional zone (JZ), and transport labyrinth zone (LZ) in either fetal sex (Figure 4C–E). However, as indicated by periodic acid Schiff staining, a maternal HFHS diet overall increased placental glycogen (Figure 4F,G). Measurement of the size of individual glycogen cells in the JZ revealed there were no differences in the average size or size frequency distribution of JZ glycogen cells in the placenta between control and HFHS diet‐fed mice (Figure 4H,J,K). Instead, a greater area of glycogen staining was related to a greater number of glycogen cells within the placenta JZ of HFHS mice (Figure 4I); an effect most pronounced in female fetuses. Using double‐label immunohistochemistry and stereology, the morphology of the LZ was also determined (Figure 4L). This showed that there was a tendency for a maternal HFHS diet to reduce the volumes of fetal capillaries (FC), trophoblasts (TB), and maternal blood space (MBS) within the LZ (Figure 4M). In addition, the surface area of the FC and MBS for substrate exchange was reduced in male but not female fetuses of HFHS dams (Figure 4N). There was no effect of a maternal HFHS diet on interhaemal membrane barrier thickness, fetal capillary length, or diameter in the placental LZ either fetal sex (Figure 4O–Q). However, there was a significant interaction of maternal diet and fetal sex on the specific diffusing capacity of the placenta, whereby it was lower in males, but higher in females of HFHS diet‐fed dams compared to their respective controls (Figure 4R).

### 2.8 Offspring survival is reduced through lactation period in pups from HFHS diet‐fed dams

In separate dams allowed to deliver naturally and continued their respective diet, we assessed impacts of a maternal HFHS diet on post‐partum body composition and offspring outcomes (up until weaning, at 3 weeks of age). Maternal body weight at weaning and change in body weight between pre‐pregnancy and weaning was not altered by the HFHS diet (Table 2). Post‐partum, HFHS diet‐fed dams had greater adiposity levels at weaning (both in g and % body weight; Table 2). However, by comparing to pre‐pregnancy adiposity levels, post‐partum HFHS dams exhibited a loss in adiposity over that period, unlike the control dams which instead showed a gain (Table 2). This pattern of change in maternal adiposity is likely due to the hyper‐mobilization of adipose depots in HFHS dams during pregnancy. Lean mass (g and %) remained less in post‐partum HFHS dams, however, the percentage gain in lean mass from pre‐pregnancy to weaning was greater in HFHS‐fed mice compared to control (∆% lean mass, *p* = 0.0061; Table 2).

**TABLE 2:** Post‐partum maternal body composition following offspring weaning, and offspring survival from birth to weaning

	Control (*N* = 31)	HFHS (*N* = 39)	*p* Value
*Post‐partum maternal body composition*			
Body weight (g)	26.16 ± 0.54	26.59 ± 0.44	NS
Delta	4.92 ± 0.50	4.70 ± 0.31	NS
Adiposity (g)	3.19 ± 0.31	5.34 ± 0.34	**<0.0001**
Delta	1.09 ± 0.30	0.20 ± 0.25	**0.0302**
Adiposity (%)	12.03 ± 0.83	19.50 ± 1.05	**<0.0001**
Delta	2.18 ± 0.85	−2.78 ± 1.03	**0.0005**
Lean (g)	19.12 ± 0.31	17.65 ± 0.28	**0.0012**
Delta	3.38 ± 0.29	3.78 ± 0.22	NS
Lean (%)	72.80 ± 0.97	66.00 ± 0.89	**<0.0001**
Delta	−0.28 ± 1.07	4.19 ± 1.13	**0.0061**
Offspring survival	**(*N* = 19, *n* = 159)**	**(*N* = 26, *n* = 181)**	
Gestation at delivery (day)	20.19 ± 0.08	20.13 ± 0.10	NS
Total litter size (D0)	8.44 ± 0.23	7.19 ± 0.43	**0.0014**
Live litter size (D0)	7.89 ± 0.48	6.65 ± 0.43	NS
Live litter size at weaning (D21)	7.56 ± 0.58	5.77 ± 0.56	**0.0370**
Pup loss by D3			
# litters with pup loss	2 (10.5%)	8 (30.8%)	NS
# pups lost	10	28	**0.0121**
% loss	6.3%	15.5%	
Pup loss by D7			
# litters with pup loss	4 (21.0%)	10 (38.5%)	NS
# pups lost per litter	13	30	**0.0307**
% loss	8.2%	16.6%	
Pup loss by D21			
# litters with pup loss	5 (26.3%)	11 (42.3%)	NS
#pups lost per litter	14	31	**0.0358**
% loss	8.8%	17.1%	
Average % loss/litter	9.1%	16.9%	NS
TLL			
# litters	1	3	NS
% litters	3.2%	11.5%	

*Note* : Post‐partum maternal body composition was determined within 1 week of weaning offspring. Data expressed at mean ± SEM unless stated otherwise and analyzed by Student's *t*‐test or Fisher's exact for continuous and categorical data, respectively; significance at *p* < 0.05. The bold values are to indicate the significant *p* values.

Abbreviations: D, postnatal day; delta, change between pre‐pregnancy and post‐weaning; *N*, number of dams; *n*, number of pups; NS, not significant; TLL, total litter loss (no pups surviving by D21).

**FIGURE 5:**
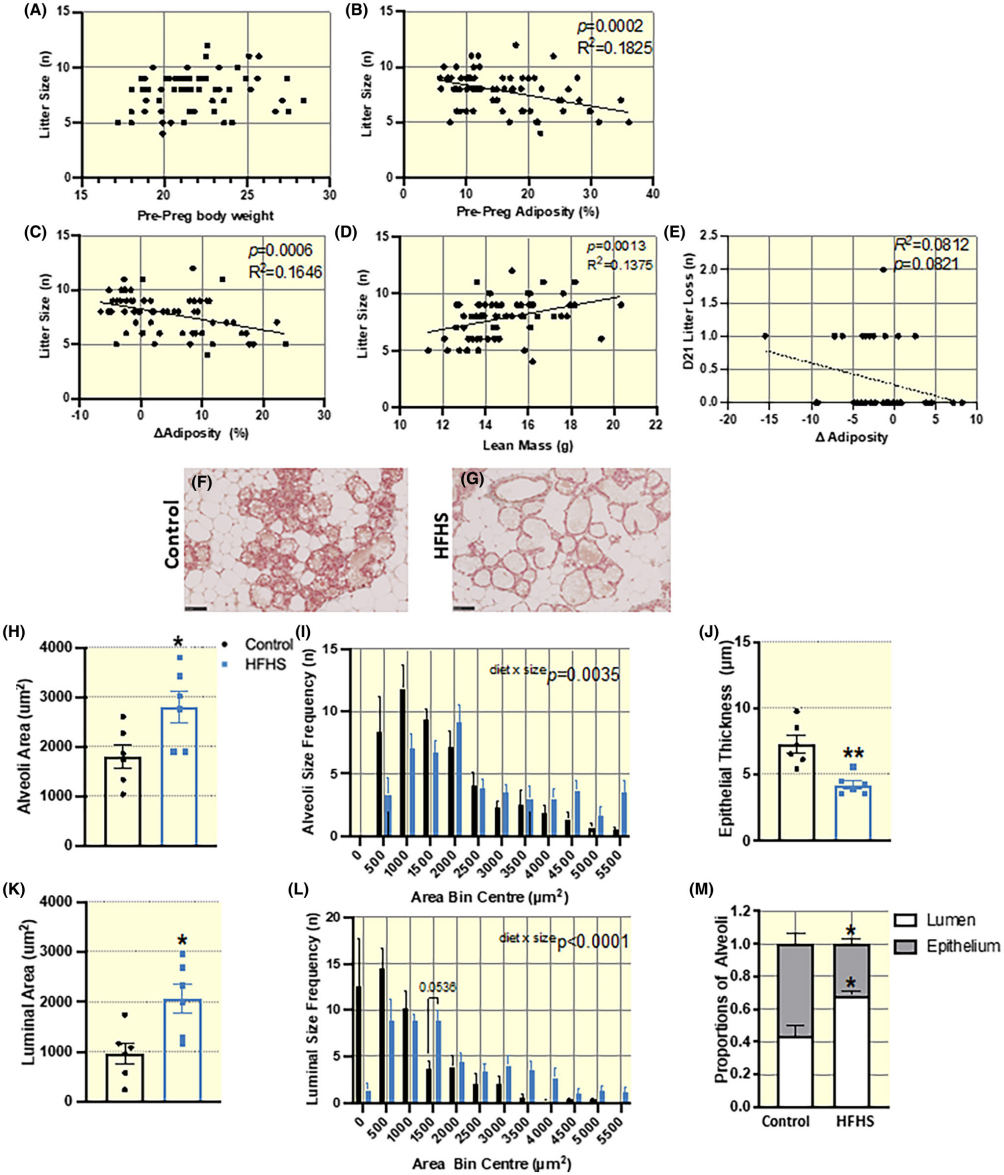
Offspring survival and lactational capacity assessed in dams fed HFHS versus control diet. (A–D) Litter size (day 0 post‐partum; D0 PP) correlated with pre‐pregnancy maternal body weight in A, pre‐pregnancy adiposity in B, change in adiposity during pregnancy in C and pre‐pregnancy lean mass in D. (E) The number of pups lost per litter at D21 PP correlated with change in maternal adiposity (%) from pre‐pregnancy to post weaning. Correlations assessed by Spearman Rank Correlation, r^2^ quoted when significant (*p* < 0.05, dietary groups combined). (F and G) Representative images of mammary tissue from control and HFHS fed dams at E18.5 of pregnancy (*n* = 6/group) at 40x (scale bar = 50 μm). (H and I) Average area and size frequency of alveoli. (J) Epithelial thickness. (K and L) Luminal area and size distribution and (M) Lipid droplet location (proximity to lumen or epithelium) in alveoli. (N−Q) Relative abundance of milk proteins in mammary tissue from control and HFHS fed dams at E18.5 of pregnancy as determined by mass spectrometry (*n* = 10/group). Data presented as individual values and/or mean ± SEM and were analyzed by *t*‐test except for frequency distributions, which were analyzed by Two‐way ANOVA (diet and size frequency).

There was no difference in gestation at delivery between HFHS and control diet‐fed dams (Table 2). Total litter size (live and dead pups) and live litter size at birth/postnatal day (D)0 and live litter size at weaning were lower in dams fed a HFHS diet compared to those fed a control diet (Table 2). There was no relationship between litter size and pre‐pregnancy body weight. However, when both dietary groups were combined, total litter size at D0 was shown to negatively correlate with maternal pre‐pregnancy adiposity and change (delta) in adiposity across pregnancy (total litter size shown in Figure 5A–C), but positively correlated with maternal pre‐pregnancy lean mass (Figure 5D). Offspring demise was significantly greater at D3 (15.5% vs 6.3%), D7 (16.6% vs 8.2%), and D21 (17.1% vs 8.8%), in litters from HFHS dams compared to control, respectively (Table 2). Average percentage loss per litter was also greater in HFHS compared to control dams (16.9% vs 9.1%). Although not significant, a greater proportion of HFHS diet‐fed dams exhibited a total litter loss by weaning as compared to control dams (11.5% vs 3.2%, respectively; Table 2). The number of pups lost by D21 showed a tendency to correlate with change in maternal adiposity between pre‐pregnancy and weaning, with a greater loss in adiposity over this period resulting in a greater litter loss by weaning (Figure 5E).

### 2.9 Mammary development during pregnancy is advanced in dams fed a HFHS diet

To gain information on the mechanisms contributing to poor offspring outcomes during lactation observed in HFHS diet‐fed dams, mammary glands were obtained from near‐term dams (E18.5), and lobuloalveolar development was assessed in hematoxylin and eosin‐stained sections using stereology (Figure 5F,G). Alveoli were larger in size (Figure 5H,I), with greater luminal area (Figure 5K,L) and reduced epithelial thickness in HFHS diet‐fed mice (Figure 5J), indicating more advanced lobuloalveolar development when compared to control diet‐fed mice. Furthermore, assessment of lipid droplet location showed lipid droplets located closer to the mammary gland epithelial luminal surface in HFHS diet‐fed mice compared to control (Figure 5M), suggesting advanced milk production and secretory capacity. The abundance of milk proteins in homogenized mammary glands as assessed by mass spectrometry revealed that HFHS diet‐fed dams had increased α‐S1‐casein, and α‐lactalbumin but decreased ɛ‐casein and H‐FABP (heart fatty acid binding protein) levels compared to control diet‐fed mice (Table 3). There were no significant differences in the other mammary gland milk proteins assessed (Table 3).

**TABLE 3:** Milk composition at E18.5

	Control (*N* = 10)	HFHS (*N* = 10)	*p* Value
*Milk composition*			
α‐S1‐casein	8359 ± 1153	14 528 ± 2012	**0.0159**
ε‐casein	0.70 ± 0.05	0.40 ± 0.03	**0.0003**
α‐lactalbumin	0.15 ± 0.04	0.35 ± 0.08	**0.0406**
H‐FABP	24.72 ± 1.85	15.97 ± 1.17	**0.0009**
b‐casein	0.60 ± 0.09	0.50 ± 0.07	NS
k‐casein	0.23 ± 0.02	0.22 ± 0.02	NS
γ‐casein	1.69 ± 0.28	2.49 ± 0.41	NS
Lactotransferrin	0.06 ± 0.02	0.03 ± 0.003	NS
Whey acidic protein	0.15 ± 0.03	0.18 ± 0.02	NS
Serum albumin	6.03 ± 1.06	4.31 ± 0.61	NS

*Note* : Data presented as mean ± SEM of peak area/mg tissue. Data were analyzed by student *t*‐test or Mann–Whitney *U* test; significance at *p* < 0.05. The bold values are to indicate the significant *p* values.

Abbreviation: NS, not significant.

## 3 DISCUSSION

This study in mice shows that a HFHS diet from 3‐weeks prior to pregnancy increases adiposity without overt impacts on body weight or fertility. Despite this, maternal metabolic physiology, and placental development during pregnancy, as well as neonatal outcome are negatively impacted in HSHF mice. In particular, the HFHS dams exhibited enhanced adipose tissue mobilization in late pregnancy in association with altered circulating lipids, and changes in key maternal metabolic organs, namely the pancreas with reduced insulin secretion and the liver with excessive fat accumulation. There was the reduced formation of placental labyrinth zone structures in both sexes, with decreased placental diffusional capacity specifically in males. Postnatally, there was a significant reduction in litter size at birth for dams fed the HFHS diet and a greater number of their pups did not survive during the lactational period. Postnatal demise seemed to be correlated to changes in maternal adiposity across the pregnancy and weaning period and aberrant development of mammary lobuloalveolar structures and milk protein production. These data are highly relevant given that women at reproductive age often consume HFHS diets and although they may not necessarily show gross alterations in body weight, these women could have metabolic problems during pregnancy with developmental consequences for their child.

It is well established that obesity negatively impacts the ability of a woman to fall pregnant. Infertility is three‐fold more common in obese women than those with a normal BMI and 15% of women seeking assisted reproductive therapies (ART) fall into the obese category.^39^, ^40^ Women with raised BMI have a longer latency to pregnancy period partly due to irregular menstrual cycles and diminished ovarian function and oocyte quality.^8^, ^41^, ^42^, ^43^ In our mouse model, we saw that a maternal HFHS diet (~3x fat and ~ 5x sugar than control diet) for 3 weeks prior to mating did not significantly increase the number of mating attempts needed to result in pregnancy or reduce the number of mice exhibiting a successful mating (copulation plug) on the first night when they were paired with a stud male. Previous studies showed that mice fed a very high‐fat diet (~6x control) exhibit altered estrous cycles only after 2 weeks on the high‐fat diet when they also show enhanced body weight.^44^ Thus, it is not surprising that no overt changes in fertility were seen in our model after 3 weeks of exposure to a high‐fat (and sugar) diet. Instead, we found that our HFHS diet‐fed dams required fewer mating attempts to achieve a pregnancy, which could suggest improved fertility compared to those fed a control diet. However, further work is required to confirm our findings and to identify the contribution of possible endocrine, immune, and metabolic alterations in the HFHS diet‐fed females prior to mating.^45^

Despite no effect of the HFHS diet on maternal body weight pre‐pregnancy, mice fed a HFHS diet showed significantly elevated adiposity. Clinically, obesity‐related risks in pregnancy and human studies of obesity are categorized by maternal BMI, not diet or maternal adiposity. BMI is used as an indicator of the adiposity component to maternal body composition, which considers weight relative to maternal height. However, BMI does not fully account for the effects of diet on body composition in the absence of overt changes in body weight, which could still lead to increased maternal adiposity and maternal metabolic alterations in pregnancy that affect the placenta and fetus. Thus, more attention should be paid to maternal diet/nutritional intake for indicating the risk of changes in maternal adiposity and metabolism that could impact on offspring outcome in their pregnancy. Studies show that even in the absence of raised maternal adiposity, an obesogenic diet can negatively impact placental structure and fetal outcome.^46^ Furthermore, women who have an obesogenic diet and may or may not be overweight in their first pregnancy, are more likely to present as obese in subsequent pregnancies.^47^, ^48^, ^49^ One paper looking at the rates of poor pregnancy outcomes in first and second pregnancies stratified risks by change in BMI units between pregnancies, found that most poor outcomes (including preeclampsia, gestational diabetes, and LGA) increased with weight gain of just one or two BMI units between pregnancies, independent of if the mother was overweight or not.^50^ Another study found that raised maternal BMI in a first pregnancy with uncomplicated outcome still increased the risk of poor outcome in the second pregnancy, even if these women have normal BMI in second pregnancy.^51^ These data together suggest there may be an effect of having a first pregnancy with sub‐optimal maternal adiposity that causes physiological adaptations leading to a higher risk of poor outcome in the subsequent pregnancies, regardless of BMI, further accentuating the importance of studying and intervening with mid‐risk (not overtly overweight or obese) women in their first pregnancies.

Interestingly, mouse dams fed a HFHS diet who had greater adiposity at the start of pregnancy showed a greater loss of adiposity across late gestation compared to controls. As a result, HFHS diet‐fed dams ended pregnancy with similar body adiposity levels to control diet‐fed dams (only weight of the retroperitoneal fat store, not renal, or gonadal was greater in HFHS dams). Compared to the anabolic state in early‐mid pregnancy, late gestation represents a catabolic state when the pregnant mother exhibits increased lipolytic activity and raised maternal free fatty acids and triglyceride levels^52^ to provide fuel for fetal growth and development, as well as maternal parturition and lactation.^53^ This catabolic state may be exaggerated in our HFHS diet‐fed mouse dams, implying increased lipolytic activity of adipose stores that results in their re‐sizing/greater loss in the lead up to term. This may also explain the increase in fat content of the liver, as circulating maternal lipids are filtered by the liver and can accumulate in this ectopic site.^38^ Circulating triglycerides and free fatty acids were both reduced in our HFHS diet‐fed dams, which has been seen previously in other similar rodent models.^38^ This could reflect a greater storage or usage of lipids by the mother, or increased utilization by the developing conceptuses. Furthermore, a less pronounced increase in triglycerides at the end of pregnancy in preparation for parturition and lactation has been seen in obese women.^52^ Future work could look at longitudinal changes in maternal lipid levels and lipid handling using lipid tolerance tests throughout pregnancy in our HFHS diet‐fed dams. This would inform on timing of the change from anabolic and catabolic states of pregnancy and whether there was a peak in circulating maternal lipids earlier in gestation (between E12.5–17.5) when maternal adiposity declined extensively in the HFHS‐fed dams. Interestingly, maternal body weight gain during pregnancy is often monitored clinically, as increased gestational weight gain is associated with an elevated risk for LGA and gestational diabetes.^54^, ^55^, ^56^ Interventions that improve maternal diet and limit maternal weight gain, therefore, have positive impacts on reducing rates of LGA and gestational diabetes.^57^ While limiting excessive gestational weight gain reduces rates of LGA in overweight/obese women, gestational weight loss is not advised due to the increased risk of delivering an SGA infant.^58^

Mice fed a HFHS diet were found to have fewer of the most abundant but small‐sized islets (20–40 μm) and reduced fasting insulin. Similar findings have been seen in other models of short‐term pre‐pregnancy obesogenic diet exposure.^59^ It is well established that during normal pregnancy, there is an increase in proliferation^60^ and size of β‐cells.^61^ There may also be β‐cell neogenesis in the mother during pregnancy.^62^ β‐Cells adaptations enable enhanced insulin production to control for the insulin resistance state of the mother during pregnancy.^29^ Our findings may therefore indicate an impaired ability of the mother to undergo β‐cell proliferation, hypertrophy, and/or islet neogenesis during pregnancy when she is consuming a HFHS diet.^29^ While dams fed the HFHS diet did not show overt alterations in insulin or glucose handling as determined by in vivo tolerance testing in late pregnancy, the subtle alterations in islet morphology likely contributed to the reduced fasted insulin and increased fed glucose levels seen and together, suggest a reduced β‐cell function in HFHS dams.^63^ Moreover, the difference in the area under the curve for the glucose tolerance test and variation between animals were large, suggesting that the lack of significance between the control and HFHS groups may have been due to limited sample size, which should be verified in future work. Prolonged exposure to high glucose levels can lead to β‐cell glucotoxicity, oxidative stress, apoptosis, and insulin insufficiency.^64^ Future work should therefore employ pancreatic islet cultures to study insulin secretion responses of the β‐cells and to dissociate the endocrine and metabolic environment from HFHS dams.

Dams fed the HFHS diet had livers with higher fat content and a greater number of large lipid droplets (8–20 μm diameter), which are indicative of steatosis^65^ and non‐alcoholic fatty liver disease (NAFLD).^66^, ^67^ This may be due to the exaggerated mobilization of adipose stores in HFHS diet‐fed dams in the second half of pregnancy and molecular changes in the liver. Previous work in rodents fed HFHS diets has reported an altered abundance of insulin signaling and lipid uptake and lipogenic proteins in the maternal liver in pregnancy.^38^ Obesity and insulin resistance are the most prevalent risk factors for hepatic steatosis, which can lead to NAFLD.^66^, ^67^ In turn, NAFLD increases the risk of gestational diabetes,^68^, ^69^ hypertensive disorders of pregnancy including, preeclampsia^70^, ^71^ and HELLP syndrome, and enhances the risk of post‐partum hemorrhage and maternal death.^71^ Further work is therefore required to understand the pathways contributing to the hepatic steatosis in our HFHS dams in pregnancy.

There was no significant effect of maternal HFHS diet consumption on fetal weight by the end of pregnancy (only non‐significant increases in the rate of SGA for males and females were observed). Work in several previous animal studies has shown that the impacts of an obesogenic diet on fetal growth vary greatly and depend on the specific nutrient composition, time of onset, and duration of the diet (recently reviewed^72^). Despite the lack of effect on fetal growth, fetal glucose, and insulin levels were both increased in response to maternal HFHS diet exposure. Placental transport and fetal glucose levels closely correlate with maternal glucose concentrations,^73^ which were also raised (in fed state) in dams fed the HFHS diet. Previous work has shown that dams fed a HFHS diet exhibit increased placental glucose transport in vivo.^74^ In addition, chronic fetal hyperglycemia has been shown to result in reduced insulin sensitivity,^75^, ^76^ diminished protein synthesis, and fetal growth restriction.^77^ These data may provide some explanation for the lack of significant changes in fetal growth despite elevated fetal glucose and insulin concentrations in our dams fed a HFHS diet. Indeed, other work has shown that fetuses of women who are obese show insulin resistance in utero.^78^

No significant differences were seen in gross placental weight or placental zone volumes between HFHS diet and control dams in either sex. However, there was a moderate reduction in the volumes of maternal blood space, trophoblast, and fetal capillaries in the placental labyrinth zone in both sexes with maternal HFHS diet exposure. Placentas of male fetuses from HFHS diet‐fed mice also showed a reduction in fetal capillary and maternal blood space surface areas and decreased diffusional capacity (a slight increase in diffusing capacity was instead seen in females). These data suggest a sexually dimorphic response of placental LZ development to a maternal HFHS diet. Junctional zone glycogen cell area and number were increased in placentas from both male and female fetuses of mice fed a HFHS diet, however, the magnitude of the effect was stronger in the females. Other work in rodent models has also reported greater effects on the junctional zone of females, compared to male counterparts.^79^ Placental glycogen is a key store of glucose, and increased glycogen stores may reflect an attempt to limit fetal exposure to glucose in hyperglycemic conditions.^80^ This may be most important given that both the HFHS diet‐fed dams and her fetuses were hyperglycemic in our study. While the definitive role of placental glycogen is elusive, the release of placental glycogen stores in late pregnancy is thought to be important for meeting placental and fetal fuel demands.^80^, ^81^, ^82^ Thus, it is possible that the increase in placental glycogen stores in HFHS diet‐fed dams may also reflect an inability to release glucose reserves with important implications for neonatal outcomes.^83^

Indeed, significant reductions in offspring survival were seen in HFHS diet‐fed dams. Despite no differences in live litter size at E18.5, there was a significant decrease in litter size at birth. Since the process of parturition was not observed, we do not know if there was an increase in peripartum stillbirth rates – as these are known to be increased by maternal obesity,^31^ or whether pups may have been cannibalized by the dam before litter size was recorded. The incidence of offspring demise continued throughout the post‐partum period, and particularly in the first 7 days.^84^ Our studies are consistent with the increased mortality seen within the first 6 days after the birth of litters born from dams with diet‐induced obesity and previous work has shown that this was not related to differences in feeding behavior or endocannabinoid systems in the offspring (important food intake and energy balance).^85^ Interestingly, previous work showed that offspring who survived/fared better in the first weeks of life were more likely to be males than females from obese mothers.^85^ As we wanted to limit handling of the neonates and minimize any effect on neonatal survival rates, we did not obtain information on sex ratio at birth or during lactation, so we do not know whether the reduction in neonatal survival was equal for both females and males. However, interestingly, the reduction in litter size and rate of litter loss was correlated with maternal percentage adiposity pre‐pregnancy and the change in adiposity between pre‐pregnancy and end of pregnancy. Furthermore, surviving litter size at weaning correlated with adiposity change between pre‐pregnancy and post‐weaning, with the greatest adiposity loss over this time resulting in greater offspring demise post‐partum.

Obese women have lower rates of breastfeeding and shorter time to breastfeeding cessation than lean counterparts.^34^ While reasons for this may be behavioral and cultural, there are data that also suggest this could be due to delayed onset of lactogenesis II (postnatal milk production).^86^ Interestingly, characteristics of more advanced mammary gland development, such as greater alveoli area, greater luminal area, thinning of epithelial wall, and migration of lipid droplets from epithelial cytoplasm to the luminal space,^87^ were seen in our HFHS diet‐fed mice. Similar results have been seen in other studies investigating mammary development in rabbits fed a high‐fat diet from pre‐pregnancy.^88^ In our study, the concentrations of key milk proteins,^89^ was altered in HFHS dams; namely α‐S1‐casein and α‐lactalbumin were increased and ɛ‐casein and H‐FABP were decreased. The physiological significance of changes in milk protein composition for offspring growth and development has been previously highlighted.^90^ However, the significance of the specific changes in milk protein levels found in our HFHS diet‐fed dams with regards to the reduced neonatal survival requires further investigation. Indeed, postnatal pup weight, other aspects of lactation, such as the ability of the mother to provide milk to her pups, maternal nurturing behavior, and the ability for pups to suckle effectively,^91^ necessitate study in future work.

It must be noted that lean mass of dams fed a HFHS diet was reduced both pre‐pregnancy and end of pregnancy, which has been seen in other mouse models fed an obesogenic diet.^92^ In our study, females were fed the HFHS diet from 6 weeks of age, which is soon after sexual maturity, and before they are fully grown. Thus, it is possible that the timing of feeding explains the lack of change in body weight and lower gain of lean mass (as well as greater reduction in adiposity during late pregnancy) in our study. Seeing as lean mass is strongly correlated with fat mass, it is difficult to distinguish the individual effects of these maternal body composition parameters on our findings. Maternal low protein diet has been known to impact fetal growth and placental function,^93^ as well as offspring health and behavior.^94^, ^95^ We are also not able to determine whether the pre‐pregnancy diet exposure and/or the pregnancy and lactation exposure to a HFHS diet was responsible for the poor neonatal outcomes in our model. Previous work using embryo transfers from high fat‐fed to a control diet‐fed mice found that pre‐gestational exposure to a high fat diet is sufficient to impact prenatal growth.^96^ However, prenatal development was also reduced in the embryos from control diet fed females implanted into high fat diet‐fed dams, and the gestational exposure to a maternal highfat diet was responsible for alterations in offspring obesity and glucose metabolism postnatally.^96^ With these important data in mind, we, therefore, are unable to determine if alterations in offspring outcomes seen in our study are due to pre, peri, or post‐natal exposure to the maternal HFHS diet.

In summary, this study uses a highly integrative approach combining in vivo imaging and metabolic testing, with morphological, biochemical, and proteomic analyses to assess the impact of a maternal western‐style HFHS diet on pregnancy physiology and lactation ability (Figure S2). In particular, mice fed a HFHS diet from 3 weeks prior to pregnancy show altered adiposity, lipid handling, metabolic organ morphology, and circulating insulin and glucose levels in pregnancy. This was associated with dysmorphic placentas, aberrant mammary gland development, and milk composition, as well as increased rates of neonatal loss. These findings may be relevant for the portion of mothers that change their culinary habits during pregnancy (short exposures to obesogenic just before and during pregnancy). Our findings also highlight the inaccuracy of relying on pre‐pregnancy maternal body weight to predict pregnancy risk of metabolic disorders that impact prenatal development and offspring health and wellbeing.

## 4 METHODS

### 4.1 Mice and husbandry

All animal experimentation was carried out under the UK Home Office Animals (Scientific Procedures) Act 1986, following ethical review by the University of Cambridge. All mice used were housed under 12:12 h dark/light photocycles with ad libitum access to water and standard rodent diet (RM3, Special Dietary Services [SDS], Witham, UK) (11% fat, 7% simple sugars of energy contribution [%kcal]) from weaning. At 6 weeks of age, female C57Bl/6J mice (who are homozygous for the *Igf2Flox* transgene but have been on a C57Bl/6J background for >10 generations, as part of a larger study and do not affect these pregnancies) were randomized to remain on the control (RM3) chow or provided with a customized high‐fat high sugar (HFHS) diet (*n* = 20/group). The HFHS diet contained high‐fat diet pellet (D12451 diet, Research Diets Inc, Denmark) and condensed milk (Carnation, Nestle, Gatwick, UK) in a 1.5: 1.0 ratio, that was blended in a food processor with water and baked into patties at 55°C for 46–48 h, as described previously.^97^ The resulting nutritional composition was 38% fat, and 33% simple sugars of energy contribution (%kcal). Mice had ad libitum access to food and water, and the HFHS diet was replaced every other day to maintain palatability.

Mouse weight gain was monitored and a TD‐NMR scanner (LF50H Minispec, Bruker, Coventry UK) was used to determine longitudinal changes in body composition (lean and fat mass) during the study, as well as to refine our study groups (Figure S1). Female mice were TD‐NMR scanned at diet assignment, 3 weeks later at time‐mating with a male (see below), as well as at E7.5, 12.5, and 17.5 of pregnancy. The subset of dams that provided litters for neonatal studies were also TD‐NMR scanned after weaning. Only control and HFHS diet‐fed mouse dams with pre‐pregnancy adiposity (% of body weight) of ≤12.4% and ≥12.5%, respectively, were mated for this study, to ensure the two groups were sufficiently separated. Using these criteria, 15 controls and 14 HFHS‐fed dams were excluded.

### 4.2 Mouse mating and fertility assessments

As briefly mentioned above, after 3 weeks on the control or HFHS diet, female mice were time‐mated with a wildtype C57Bl/6J proven stud male. Females were with a male for no more than 5 days or until copulation plug was seen. Mice were then weighed to assess if pregnant (regardless of whether a plug was found) and then re‐mated if not pregnant. The presence of a copulation plug was considered E0.5. Females on HFHS diet had access to control diet while housed with males for time‐mating only. Female mice who had plugged but did not result in a pregnancy were re‐mated for a maximum of three attempts. Female fecundity was noted by tracking plug rates, time to plug and pregnancy rates (proportion of mating attempts that resulted in pregnancy). Mating attempts equal the number of times a female was put with a male until a pregnancy resulted.

A subset of pregnant control and HFHS mice underwent either metabolic testing at E17.5 with terminal collection of maternal and feto‐placental tissues at E18.5 or were allowed to litter and attend to their young for a 21‐day lactation period (Figure 1). For the latter cohort, the dams remained on their respective diet and gestational age at delivery, litter size, and offspring death/survival during the lactation period were recorded.

### 4.3 Maternal glucose and insulin handling in pregnancy

Mice at E17.5 were fasted from 10:00 am for 4 h and either underwent an insulin tolerance test (ITT) or glucose tolerance test (GTT) (*n* = 5/group/test) by administering an intraperitoneal bolus dose of 0.25 U/kg insulin (Actrapid, Henry Schein, UK) or 1 g/kg of glucose (Dechra Veterinary Products, UK), respectively. Serial blood sampling was done through a tail nick at 0, 15, 30, 45, 60, and 120 min to measure blood glucose using a OneTouch Select Plus® glucose meter (Lifescan, Uxbridge, UK). During the GTT, maternal blood samples collected at 0 and 15 min were centrifuged at 3000 rpm for 10 min to collect serum for determining maternal fasted and glucose‐stimulated insulin concentrations. Insulin concentrations were determined using Mouse Insulin ELISA (Mercodia, Sweden) following the manufacturer's protocols. Insulin concentrations were calculated interpolated from a standard curve using cubic spline regression in Prism (USA, average intra‐assay %CV 3.89).

### 4.4 Pregnancy outcome indicators and collection of maternal tissues

After the ITT or GTT, dams were immediately returned to a cage for ad libitum access to their respective diet (control or HFHS). The following morning (E18.5), dams were either anesthetized (1:1:0.16 Fentanyl (Fentadon, Dechra Veterinary Products UK), Midazolam (Hypnovel®, Roche, UK), and Ketamine (Ketavet, Zoetis, UK) diluted in 3.26 parts water and injected i.p. with 10 μl/g) for collection of cardiac blood into an EDTA tube (GTT animals), or immediately schedule 1 killed by cervical dislocation (ITT animals). Fetuses were removed by laparotomy and decapitated. Fetal blood was also collected from the animals that had undergone a GTT the previous day. Fetuses were sexed by eye and sex was then confirmed by SRY genotyping. The male and female pup with the closest weight to the average for the litter per sex was selected and fetal blood was collected immediately following decapitation. Maternal and fetal blood were centrifuged at 12 000 rpm to obtain plasma and serum fractions, respectively, and samples were then stored at −20°C until analysis. Maternal organs and fetuses and placentas were weighed. From a sub‐set of mice, maternal liver, pancreas, and right abdominal mammary gland, and placenta per sex per litter were fixed in 4% paraformaldehyde (PFA) and processed into paraffin wax for sectioning and histological analysis. From a different subset of mice, maternal liver, and left abdominal mammary gland was snap frozen and stored at −80°C for biochemical analysis.

Pregnancy outcome was assessed by total litter size, number of viable fetuses, and presence of resorptions within the litter, fetal, and placental weight, and fetal weight distribution using <5th, 5–95th, and >95th centiles of the control diet group as clinically relevant markers of small for gestational age (SGA), appropriate for gestational age (AGA) and large for gestational age (LGA). Fetal brain and liver weights and blood glucose were also determined from a subset of fetuses within each litter.

### 4.5 Plasma and serum analysis

Maternal plasma and fetal serum samples were analyzed by the Core Biochemical Assay Laboratory (CBAL, Cambridge University Hospital NHS Foundation Trust) to determine the concentrations of insulin (Multiplex ElectroChemical Luminescence Immunoassay, MesoScale Discovery, USA, inter‐assay %CV 4.4–4.7%), triglycerides (Lipase Enzyme Assay, Siemens Healthcare, Germany, inter‐assay %CV 3.4–5.5%), and non‐esterified free fatty acids (maternal plasma only, Roche Free Fatty Acid Kit (Sigma‐Aldrich, inter‐assay %CV 15.0–12.5%)).

### 4.6 Maternal liver assessments

#### 4.6.1 Fat content assay

Maternal liver fat content was assessed in 100 mg of tissue homogenized in 1 ml Folch mixture (chloroform:methanol 2:1), mixed with water and centrifuged at 13 200 rpm for 10 min to obtain the lipid phase. The weight of lipid content was assessed by dry extracting fats at 37°C overnight and presented as a percentage of wet tissue mass.^38^

#### 4.6.2 Glycogen content assay

Glycogen content of the maternal liver was assessed as previously described.^74^ In brief, 100 mg/ml of tissue in distilled H_2_O (dH_2_O) was incubated at 55°C for 10 min with acetate buffer, then a further 10 min with or without 70 U/mg amyloglucosidase enzyme in duplicate. 0.3 M zinc sulfate and 0.3 M barium hydroxide (Sigma‐Aldrich) were added to de‐proteinise the samples. After centrifugation at 3000 rpm for 10 mins, glucose content of the supernatant was quantified on a YSI Analyzer (YSI Inc., USA). Glucose concentrations were compared between assays in the presence and absence of the amyloglucosidase to quantify the conversion of glycogen to glucose as the indicator of glycogen content and presented as mg/g of wet liver tissue.

#### 4.6.3 Liver steatosis

Hematoxylin and eosin (H&E) stained 7 μm sections of the left lateral lobe of maternal livers were imaged in a systematic, random fashion to obtain ten 20x images representative of the whole tissue. Images were processed using ImageJ (NIH Image, USA) and a developed macro that had been previously validated,^65^ to assess lipid droplets size as a marker of steatosis. Briefly, the original H&E image was converted to 8‐bit gray‐scale image, color inverted, and a threshold of 150–255 gray levels applied to identify the lipid droplets. Particle analysis was then configured to count particles with circularity between 0.5 and 1.0 AU and a diameter between 0.1 and 50 μm. The macros created a database with each droplet diameter and area in each image, which were compiled for the 10 images per sample from which actual and relative distributions of lipid droplet size could be assessed.

### 4.7 Maternal pancreas assessments

#### 4.7.1 Islet morphology by immunohistochemistry

Three non‐adjacent 5 μm sections (100 μm apart in depth) of paraffin‐embedded maternal pancreas were assayed to ensure sampling throughout the entire tissue was stained by standard immunohistochemical techniques. Briefly, re‐hydrated sections were treated with 10% hydrogen peroxide (Fisher Chemical, USA) in methanol for 15 min to quench endogenous peroxidases. Antigen retrieval was conducted in citrate buffer (pH 6.0) in an 80°C water bath for 30 min. Sections were then blocked using blocking buffer (2% BSA, 1% skimmed milk powder, 0.1% Tween in dH_2_O) including 10% goat serum for 1 h. Sections were then incubated overnight at 4°C with either rabbit anti‐mouse insulin (1:1000, #4590 Cell Signaling) diluted in the blocking buffer. Bound antibody was detected using biotinylated goat anti‐rabbit secondary (1:1000, ab6720, Abcam) and then streptavidin‐horseradish peroxidase (1:500, S000‐03, Rockland Immunochemicals) each for 1 h at room temperature. Bound antibody was then visualized using 3, 3′‐diaminobenzidine (DAB, Sigma) and slides were counterstained with Nuclear Fast Red (Vector Laboratories). Washes throughout the procedure were done with Tris‐Buffered Saline (TBS) or TBS‐tween (0.05%) where appropriate. Whole sections were imaged by Nanozoom slide scanner (Hamamatsu, Japan). Insulin‐stained sections were analyzed for β‐cell and islet area/diameter by manually drawing around insulin‐positive stained areas. β‐Cell mass was presented as % area and mass in mg by multiplying percentage area by total pancreatic wet weight.

### 4.8 Maternal mammary gland assessments

#### 4.8.1 Mammary gland histology

Three non‐adjacent 5 μm mammary gland sections per sample were stained with Masson's Trichrome to stain collagen and muscle fibers. Samples were deparaffinized and hydrated. Sections were placed in Bouin's Fluid at 56°C for 1 h, then in working Weigert's iron hemoxylin stain for 10 min followed by 10 min in Biebrich Scarlet‐Acid Fuchsin Solution and 5 min in phosphotungstic‐phosphomolybdic acid solution. Finally, samples were stained in aniline blue stain solution for 10 min, placed in acetic acid for 1 min and dehydrated. Once scanned by Nanozoom slide scanner (Hamamatsu, Japan), ten fields of view captured at 20x magnification from representative areas of each section were analyzed by Image J (NIH Image). For mammary gland area, an ImageJ macro was developed. Briefly, scale was adjusted to 2.22 pixels/μm. The picture was converted to 8‐bit and a threshold with cut‐off values of 0–215 gray levels was applied. Alveolar development was analyzed on single mid‐tissue sections per sample. Using NDP view, alveolar and luminal area/diameter were quantified by manually drawing around 50 randomly selected alveoli (5 per lobule over 10 lobules) per section at 80x magnification. Mean epithelial thickness was calculated as; alveoli diameter – luminal diameter. Average area of cytoplasmic lipid droplets (CLD) was analyzed using Adobe Photoshop CC 2017 of ten 80x magnification images from single sections per sample and was presented as both a mean and a frequency distribution and location in alveoli (epithelial/luminal adjacent/luminal).

#### 4.8.2 Characterization of mammary tissue milk proteins

Mammary tissue was powered and then 13‐20 mg was homogenized in 250 μl of 6 M guanidine hydrochloride (Sigma‐Aldrich) with Lysing Matrix D (MPbio) in a FastPrep‐24 homogenizer. Proteins were extracted by adding 75% (v/v) acetonitrile (ACN) (Fisher Scientific) to 150 μl of lysate, vortexing to mix, and centrifuged (12 000 g, 10 min, 4°C) to obtain the aqueous phase. The aqueous phase was collected and dried under oxygen‐free nitrogen (40°C) on a SPE Dry evaporator system (Biotage, Upsalla, Sweden). Extracts were re‐suspended in 500 μl of 0.1% (v/v) formic acid in water and loaded to a Waters HLB μElution solid‐phase extraction plate (Waters, Milford, MA) as described previously.^98^ Following reduction and alkylation, 10 μl trypsin (100 μg/ml) was added and incubated overnight at 37°C. The following day, 20 μl 1% formic acid was added.

LC–MS/MS was performed on a M‐Class UPLC system (Waters, Milford, USA) with a TQ‐XS triple quadrupole mass spectrometer with an IonKey interface (Waters). Digested sample (5 μl) was loaded onto a NanoEase 0.3 × 50 mm, BEH C18 trap column (Waters), at 15 μl/min for 3 min. Peptides were then separated on a 0.15 × 100 mm HSS T3 iKey (Waters) at 3 μl/min. The load and initial conditions were 90% A (0.1% formic acid in water v/v) and 10% B (0.1 %Formic acid in ACN v/v) rising to 55% B over 13 min, before a 3‐min flush at 85% B and returning to initial conditions for a 20‐min run time. Peptide transitions that were used to monitor for selected proteins are included in Table S2. Peak areas for the proteins were generated in the TargetLynx XS program (Waters). The whole procedure was performed by the Peptidomics and Proteomics Core at the Wellcome‐MRC Institute of Metabolic Sciences, Metabolic Research Laboratories.

### 4.9 Placental assessments

#### 4.9.1 Gross placental structure

Paraffin‐embedded placentas were exhaustively and serially sectioned at 8 μm with one section in every 20 mounted onto slides for H&E staining. Nanozoomed scans of stained sections were used to assess gross placental structure using ImageJ and point counting to determine the volume density/proportion of decidua basalis (DB), junctional zone (JZ), or labyrinth zone (LZ).

#### 4.9.2 Analysis of placental junctional zone glycogen cells

Periodic acid Schiff staining was performed on one 5 μm mid‐sagittal section of each placenta. Rehydrated sections were incubated in Periodic Acid solution for 5 min at RT, rinsed in distilled water, and then immersed in Schiff's reagent for 15 min at RT. Sections were counterstained with hematoxylin (10 min). Mounted sections were scanned and then NDP.view was used to draw around 100 (25/edge, 50/central) randomly selected glycogen cells within the JZ. The distribution of glycogen cell area and total area of glycogen cells in the JZ was then calculated.

#### 4.9.3 Placental labyrinth zone morphology

Cytokeratin and isolectin dual staining was performed on one 5 μm mid‐sagittal section per placenta as described previously.^99^ In brief, following rehydration and blocking of endogenous peroxidase activity, antigen retrieval was conducted in 0.04% Pepsin in 0.01 M HCl at 37°C for 10 min then blocked with blocking buffer including 10% goat serum (as described above for the pancreas immunohistochemistry). A biotinylated antibody against lectin (Isolectin B4, B‐1205, Vector Laboratories, 1:250) was applied for 90 min at 37°C and visualized using streptavidin and DAB. Subsequently, sections were re‐blocked with blocking buffer including 10% goat serum and then a rabbit anti‐pan cytokeratin antibody (1:75, nb600‐549, Novus Biologicals) was added to each section and incubated overnight at 4°C. Sections were incubated in goat anti‐rabbit antibody‐alkaline phosphatase secondary antibody for 1 h at room temperature then developed using NBT/BCIP (Thermofisher) in the dark. Sections were counterstained with Nuclear fast red, then dehydrated and mounted for imaging as described above. Sections were analyzed in NDP.view using random systematic sampling as described previously.^99^, ^100^

### 4.10 Statistics

Data were analyzed using GraphPad Prism (La Jolla, USA). Temporal maternal adiposity and blood glucose concentrations were analyzed by Two‐way ANOVA with multiple comparisons. Area above the curve (AAC) or area under the curve (AUC) was calculated for temporal blood glucose concentrations during ITT and GTTs, respectively. The distribution of data was assessed by D'Agostino & Pearson test. When data showed normal distribution it was analyzed by student *t*‐test or Two‐way ANOVA, as appropriate. When data were not normally distributed, it was analyzed Mann–Whitney *U* test. Correlations were determined by the Spearman‐rank test. Raw distributions were initially assessed by Kolmogorov–Smirnov test. The 5th and 95th percentiles of fetal weight from control populations were calculated using Z critical values of −1.649 and 1.649, respectively using the formula: (Z critical value × standard deviation) + mean for either whole population or separated sexes where appropriate. Proportion of population categorized as SGA or LGA was analyzed by Fisher's Exact test. Where multiple values for one parameter were obtained in the litter (namely fetal and placental weights), the closest to average per sex per litter was used in statistical analysis. All data were considered significant at *p* < 0.05, and a statistical tendency at *p* < 0.1.

## FUNDING INFORMATION

This work was funded by the Medical Research Council New Investigator Grant (MR/R022690/1/RG93186) and Lister Institute of Preventative Medicine Research Prize (RG39692) to ANS‐P. GCLL was supported by a Society for Endocrinology Summer Studentship. AAC was supported by CONICYT PFCHA/DOCTORADO NACIONAL/2019–21 190 352. The LC–MS/MS proteomics work was performed at the Wellcome Trust MRC Institute of Metabolic Science and was supported by the Wellcome Trust grants (106 262/Z/14/Z, 106263/Z/14/Z), MRC grants (MRC_MC_UU_12012/3 and MRC‐enhancing UK clinical research grant MR/M009041/1) and the Cambridge Biomedical Research Centre (NIHR‐BRC Gastrointestinal Diseases theme). This work was also supported by the MRC MDU Mouse Biochemistry Laboratory (Core Biochemical Assay Laboratory, Cambridge) [MC_UU_00014/5].

## CONFLICT OF INTEREST

The authors declare that no conflicts of interest exist.

## Supporting information


Supinfo

Figure S1

Figure S2

## References

[1] Kopp W . How western diet and lifestyle drive the pandemic of obesity and civilization diseases. Diabetes Metab Syndr Obes. 2019;12:2221‐2236. doi:10.2147/DMSO.S216791 31695465 PMC6817492

[2] Balanza R , García‐Lorda P , Pérez‐Rodrigo C , Aranceta J , Bonet MB , Salas‐Salvadó J . Trends in food availability determined by the food and agriculture Organization's food balance sheets in Mediterranean Europe in comparison with other European areas. Public Health Nutr. 2007;10(2):168‐176. doi:10.1017/S1368980007246592 17261226

[3] Temme E , Huybrechts I , Vandevijvere S , et al. Energy and macronutrient intakes in Belgium: results from the first National Food Consumption Survey. Br J Nutr. 2010;103(12):1823‐1829. doi:10.1017/S0007114510000085 20187986

[4] Austin GL , Ogden LG , Hill JO . Trends in carbohydrate, fat, and protein intakes and association with energy intake in normal‐weight, overweight, and obese individuals: 1971–2006. Am J Clin Nutr. 2011;93(4):836‐843. doi:10.3945/ajcn.110.000141 21310830

[5] Hillemeier MM , Weisman CS , Chuang C , Downs DS , McCall‐Hosenfeld J , Camacho F . Transition to overweight or obesity among women of reproductive age. J Womens Health. 2011;20(5):703‐710. doi:10.1089/jwh.2010.2397 PMC3096512 21599427

[6] World Health Organisation Obesity and Overweight Fact Sheet (updated 2016) . https://www.who.int/en/news‐room/fact‐sheets/detail/obesity‐and‐overweight

[7] Denison FC , Aedla NR , Keag O , et al. Care of women with obesity in pregnancy: Green‐top guideline no. 72. BJOG. 2019;126(3):e62‐e106. doi:10.1111/1471-0528.15386 30465332

[8] Brewer CJ , Balen AH . The adverse effects of obesity on conception and implantation. Reproduction. 2010;140(3):347‐364. doi:10.1530/REP-09-0568 20395425

[9] Marchi J , Berg M , Dencker A , Olander EK , Begley C . Risks associated with obesity in pregnancy, for the mother and baby: a systematic review of reviews. Obes Rev. 2015;16(8):621‐638. doi:10.1111/obr.12288 26016557

[10] Stubert J , Reister F , Hartmann S , Janni W . The risks associated with obesity in pregnancy. Dtsch Arztebl Int. 2018;115:276‐283. doi:10.3238/arztebl.2018.0276 29739495 PMC5954173

[11] Shankar K , Harrell A , Liu X , Gilchrist JM , Ronis MJJ , Badger TM . Maternal obesity at conception programs obesity in the offspring. Am J Physiol Regul Integr Comp Physiol. 2008;294(2):R528‐R538. doi:10.1152/ajpregu.00316.2007 18032473

[12] Lahti‐Pulkkinen M , Bhattacharya S , Wild SH , et al. Consequences of being overweight or obese during pregnancy on diabetes in the offspring: a record linkage study in Aberdeen, Scotland. Diabetologia. 2019;62(8):1412‐1419. doi:10.1007/s00125-019-4891-4 31214738 PMC6647186

[13] Freeman DJ . Effects of maternal obesity on fetal growth and body composition: implications for programming and future health. Semin Fetal Neonatal Med. 2010;15(2):113‐118. doi:10.1016/j.siny.2009.09.001 19853544

[14] Morgan KL , Rahman MA , Macey S , et al. Obesity in pregnancy: a retrospective prevalence‐based study on health service utilisation and costs on the NHS. BMJ Open. 2014;4(2):e003983. doi:10.1136/bmjopen-2013-003983 PMC3939655 24578535

[15] Koning AMH , Kuchenbecker WKH , Groen H , et al. Economic consequences of overweight and obesity in infertility: a framework for evaluating the costs and outcomes of fertility care. Hum Reprod Update. 2010;16(3):246‐254. doi:10.1093/humupd/dmp053 20056674

[16] Chen C , Xu X , Yan Y . Estimated global overweight and obesity burden in pregnant women based on panel data model. PLoS ONE. 2018;13(8):e0202183. doi:10.1371/journal.pone.0202183 30092099 PMC6084991

[17] Sam S . Obesity and polycystic ovary syndrome. Obes Manag. 2007;3(2):69‐73. doi:10.1089/obe.2007.0019 20436797 PMC2861983

[18] Robker RL . Evidence that obesity alters the quality of oocytes and embryos. Pathophysiology. 2008;15(2):115‐121. doi:10.1016/j.pathophys.2008.04.004 18599275

[19] Snider AP , Wood JR . Obesity induces ovarian inflammation and reduces oocyte quality. Reproduction. 2019;158(3):R79‐R90. doi:10.1530/REP-18-0583 30999278

[20] Bellver J , Melo MAB , Bosch E , Serra V , Remohí J , Pellicer A . Obesity and poor reproductive outcome: the potential role of the endometrium. Fertil Steril. 2007;88(2):446‐451. doi:10.1016/j.fertnstert.2006.11.162 17418840

[21] Bellver J , Martínez‐Conejero JA , Labarta E , et al. Endometrial gene expression in the window of implantation is altered in obese women especially in association with polycystic ovary syndrome. Fertil Steril. 2011;95(7):2335‐2341.e8. doi:10.1016/j.fertnstert.2011.03.021 21481376

[22] Rhee JS , Saben JL , Mayer AL , et al. Diet‐induced obesity impairs endometrial stromal cell decidualization: a potential role for impaired autophagy. Hum Reprod. 2016;31(6):1315‐1326. doi:10.1093/humrep/dew048 27052498 PMC4871191

[23] Metwally M , Ong KJ , Ledger WL , Li TC . Does high body mass index increase the risk of miscarriage after spontaneous and assisted conception? A meta‐analysis of the evidence. Fertil Steril. 2008;90(3):714‐726. doi:10.1016/j.fertnstert.2007.07.1290 18068166

[24] Lashen H , Fear K , Sturdee DW . Obesity is associated with increased risk of first trimester and recurrent miscarriage: matched case‐control study. Hum Reprod. 2004;19(7):1644‐1646. doi:10.1093/humrep/deh277 15142995

[25] Hershman M , Mei R , Kushner T . Implications of nonalcoholic fatty liver disease on pregnancy and maternal and child outcomes. Gastroenterol Hepatol. 2019;15(4):221‐228. PMC6696596 31435201

[26] Catalano PM , Shankar K . Obesity and pregnancy: mechanisms of short term and long term adverse consequences for mother and child. BMJ. 2017;356:j1. doi:10.1136/bmj.j1 28179267 PMC6888512

[27] Sferruzzi‐Perri AN , Lopez‐Tello J , Napso T , Yong HEJ . Exploring the causes and consequences of maternal metabolic maladaptations during pregnancy: lessons from animal models. Placenta. 2020;98:43‐51. doi:10.1016/j.placenta.2020.01.015 33039031 PMC7548399

[28] Napso T , Yong HEJ , Lopez‐Tello J , Sferruzzi‐Perri AN . The role of placental hormones in mediating maternal adaptations to support pregnancy and lactation. Front Physiol. 2018;9:1091. doi:10.3389/fphys.2018.01091 30174608 PMC6108594

[29] Salazar‐Petres ER , Sferruzzi‐Perri AN . Pregnancy‐induced changes in β‐cell function: what are the key players? J Physiol. 2022;600(5):1089‐1117. 33704799 10.1113/JP281082

[30] Fowden AL , Camm EJ , Sferruzzi‐Perri AN . Effects of maternal obesity on placental phenotype. Curr Vasc Pharmacol. 2021;19(2):113‐131. 32400334 10.2174/1570161118666200513115316

[31] Radulescu L , Munteanu O , Popa F , Cirstoiu M . The implications and consequences of maternal obesity on fetal intrauterine growth restriction. J Med Life. 2013;6(3):292‐298. 24155784 PMC3806033

[32] Lewandowska M . Maternal obesity and risk of low birth weight, fetal growth restriction, and macrosomia: multiple analyses. Nutrients. 2021;13(4):1213. 33916963 10.3390/nu13041213 PMC8067544

[33] Leddy MA , Power ML , Schulkin J . The impact of maternal obesity on maternal and fetal health. Rev Obstet Gynecol. 2008;1(4):170‐178. 19173021 PMC2621047

[34] Bever Babendure J , Reifsnider E , Mendias E , Moramarco MW , Davila YR . Reduced breastfeeding rates among obese mothers: a review of contributing factors, clinical considerations and future directions. Int Breastfeed J. 2015;10(1):21. 26140049 10.1186/s13006-015-0046-5 PMC4488037

[35] Plaza‐Díaz J , Fontana L , Gil A . Human milk oligosaccharides and immune system development. Nutrients. 2018;10(8):1038. 30096792 10.3390/nu10081038 PMC6116142

[36] Dearden L , Buller S , Furigo IC , Fernandez‐Twinn DS , Ozanne SE . Maternal obesity causes fetal hypothalamic insulin resistance and disrupts development of hypothalamic feeding pathways. Molecular Metabolism. 2020;42:101079. doi:10.1016/j.molmet.2020.101079 32919096 PMC7549144

[37] Mahany EB , Han X , Borges BC , et al. Obesity and high‐fat diet induce distinct changes in placental gene expression and pregnancy outcome. Endocrinology. 2018;159(4):1718‐1733. doi:10.1210/en.2017-03053 29438518 PMC6456933

[38] Musial B , Vaughan OR , Fernandez‐Twinn DS , et al. A Western‐style obesogenic diet alters maternal metabolic physiology with consequences for fetal nutrient acquisition in mice. J Physiol. 2017;595(14):4875‐4892. 28382681 10.1113/JP273684 PMC5509867

[39] Lintsen AME , Pasker‐de Jong PCM , de Boer EJ , et al. Effects of subfertility cause, smoking and body weight on the success rate of IVF. Hum Reprod. 2005;20(7):1867‐1875. doi:10.1093/humrep/deh898 15817580

[40] Wilkes S , Murdoch A . Obesity and female fertility: a primary care perspective. J Fam Plann Reprod Health Care. 2009;35(3):181‐185. doi:10.1783/147118909788707995 19622210

[41] Pandey S , Bhattacharya S . Impact of obesity on gynecology. Womens Health (Lond). 2010;6(1):107‐117. doi:10.2217/whe.09.77 20088734

[42] Wei S , Schmidt MD , Dwyer T , Norman RJ , Venn AJ . Obesity and menstrual irregularity: associations with SHBG, testosterone, and insulin. Obesity. 2009;17(5):1070‐1076. doi:10.1038/oby.2008.641 19180069

[43] van der Steeg JW , Steures P , Eijkemans MJC , et al. Obesity affects spontaneous pregnancy chances in subfertile, ovulatory women. Hum Reprod. 2008;23(2):324‐328. doi:10.1093/humrep/dem371 18077317

[44] Chakraborty TR , Donthireddy L , Adhikary D , Chakraborty S . Long‐term high fat diet has a profound effect on body weight, hormone levels, and estrous cycle in mice. Med Sci Monit. 2016;22:1601‐1608. doi:10.12659/MSM.897628 27171231 PMC4917314

[45] Goldsammler M , Merhi Z , Buyuk E . Role of hormonal and inflammatory alterations in obesity‐related reproductive dysfunction at the level of the hypothalamic‐pituitary‐ovarian axis. Reprod Biol Endocrinol. 2018;16(1):45. doi:10.1186/s12958-018-0366-6 29743077 PMC5941782

[46] Frias AE , Morgan TK , Evans AE , et al. Maternal high‐fat diet disturbs uteroplacental hemodynamics and increases the frequency of stillbirth in a nonhuman primate model of excess nutrition. Endocrinology. 2011;152(6):2456‐2464. doi:10.1210/en.2010-1332 21447636 PMC3100625

[47] Bogaerts A , van den Bergh BRH , Ameye L , et al. Interpregnancy weight change and risk for adverse perinatal outcome. Obstet Gynecol. 2013;122:999‐1009. doi:10.1097/AOG.0b013e3182a7f63e 24104777

[48] Nartea R , Mitoiu BI , Nica AS . Correlation between pregnancy related weight gain, postpartum weight loss and obesity: a prospective study. J Med Life. 2019;12(2):178‐183. doi:10.25122/jml-2019-0015 31406521 PMC6685304

[49] Hoff GL , Cai J , Okah FA , Dew PC . Pre‐pregnancy overweight status between successive pregnancies and pregnancy outcomes. J Womens Health. 2009;18(9):1413‐1417. doi:10.1089/jwh.2008.1290 19698074

[50] Villamor E , Cnattingius S . Interpregnancy weight change and risk of adverse pregnancy outcomes: a population‐based study. Lancet. 2006;368(9542):1164‐1170. doi:10.1016/S0140-6736(06)69473-7 17011943

[51] Tabet M , Flick LH , Tuuli MG , Macones GA , Chang JJ . Prepregnancy body mass index in a first uncomplicated pregnancy and outcomes of a second pregnancy. Am J Obstet Gynecol. 2015;213(4):548.e1‐548.e7. doi:10.1016/j.ajog.2015.06.031 26103529

[52] Herrera E . Metabolic adaptations in pregnancy and their implications for the availability of substrates to the fetus. Eur J Clin Nutr. 2000;54:S47‐S51. doi:10.1038/sj.ejcn.1600984 10805038

[53] Qureshi IA , Xi XR , Limbu YR , Bin HY , Chen MIC . Hyperlipidaemia during normal pregnancy, parturition and lactation. Ann Acad Med Singap. 1999;28(2):217‐221. 10497670

[54] Liu Z , Ao D , Yang HX , Wang Y . Gestational weight gain and risk of gestational diabetes mellitus among Chinese women. Chin Med J. 2014;127(7):1255‐1260. doi:10.3760/cma.j.issn.0366-6999.20132772 24709176

[55] Black MH , Sacks DA , Xiang AH , Lawrence JM . The relative contribution of prepregnancy overweight and obesity, gestational weight gain, and IADPSG‐defined gestational diabetes mellitus to fetal overgrowth. Diabetes Care. 2013;36(1):56‐62. doi:10.2337/dc12-0741 22891256 PMC3526206

[56] Weschenfelder F , Lehmann T , Schleussner E , Groten T . Gestational weight gain particularly affects the risk of large for gestational age infants in non‐obese mothers. Geburtshilfe Frauenheilkd. 2019;79(11):1183‐1190. doi:10.1055/a-0891-0919 31736507 PMC6846725

[57] Vesco KK , Karanja N , King JC , et al. Efficacy of a group‐based dietary intervention for limiting gestational weight gain among obese women: a randomized trial. Obesity. 2014;22(9):1989‐1996. doi:10.1002/oby.20831 25164259 PMC4407817

[58] Kapadia MZ , Park CK , Beyene J , Giglia L , Maxwell C , McDonald SD . Weight loss instead of weight gain within the guidelines in obese women during pregnancy: a systematic review and meta‐analyses of maternal and infant outcomes. PLoS ONE. 2015;10(7):e0132650. doi:10.1371/journal.pone.0132650 26196130 PMC4509670

[59] Pennington KA , van der Walt N , Pollock KE , Talton OO , Schulz LC . Effects of acute exposure to a high‐fat, high‐sucrose diet on gestational glucose tolerance and subsequent maternal health in mice. Biol Reprod. 2017;96(2):435‐445. doi:10.1095/biolreprod.116.144543 28203773

[60] Karnik SK , Chen H , McLean GW , et al. Menin controls growth of pancreatic ‐cells in pregnant mice and promotes gestational diabetes mellitus. Science. 2007;318(5851):806‐809. doi:10.1126/science.1146812 17975067

[61] Huang C , Snider F , Cross JC . Prolactin receptor is required for Normal glucose homeostasis and modulation of β‐cell mass during pregnancy. Endocrinology. 2009;150(4):1618‐1626. doi:10.1210/en.2008-1003 19036882

[62] Hill DJ . Placental control of metabolic adaptations in the mother for an optimal pregnancy outcome. What goes wrong in gestational diabetes? Placenta. 2018;69:162‐168. 29352600 10.1016/j.placenta.2018.01.002

[63] Cerf ME . Beta cell dysfunction and insulin resistance. Front Endocrinol. 2013;4:37. doi:10.3389/fendo.2013.00037 PMC3608918 23542897

[64] Bensellam M , Laybutt DR , Jonas J‐C . The molecular mechanisms of pancreatic β‐cell glucotoxicity: recent findings and future research directions. Mol Cell Endocrinol. 2012;364(1–2):1‐27. doi:10.1016/j.mce.2012.08.003 22885162

[65] Piao D , Ritchey JW , Holyoak GR , et al. *In vivo*percutaneous reflectance spectroscopy of fatty liver development in rats suggests that the elevation of the scattering power is an early indicator of hepatic steatosis. J Innov Opt Health Sci. 2018;11(4):1850019. doi:10.1142/S1793545818500190

[66] Arisqueta L , Navarro‐Imaz H , Labiano I , Rueda Y , Fresnedo O . High‐fat diet overfeeding promotes nondetrimental liver steatosis in female mice. Am J Physiol Gastrointest Liver Physiol. 2018;315(5):G772‐G780. doi:10.1152/ajpgi.00022.2018 30095299

[67] Cohen JC , Horton JD , Hobbs HH . Human fatty liver disease: old questions and new insights. Science. 2011;332(6037):1519‐1523. doi:10.1126/science.1204265 21700865 PMC3229276

[68] Azzaroli F , Mazzella G , Marchesini G , Brodosi L , Petroni ML . Fatty liver in pregnancy: a narrative review of two distinct conditions. Expert Rev Gastroenterol Hepatol. 2020;14(2):127‐135. doi:10.1080/17474124.2020.1715210 31928239

[69] Lee YW , Yarrington CD . Obstetric outcomes in women with nonalcoholic fatty liver disease. Metab Syndr Relat Disord. 2017;15(8):387‐392. doi:10.1089/met.2017.0058 28846061

[70] Hagström H , Höijer J , Ludvigsson JF , et al. Adverse outcomes of pregnancy in women with non‐alcoholic fatty liver disease. Liver Int. 2016;36(2):268‐274. doi:10.1111/liv.12902 26114995

[71] Sarkar M , Grab J , Dodge JL , et al. Non‐alcoholic fatty liver disease in pregnancy is associated with adverse maternal and perinatal outcomes. J Hepatol. 2020;73(3):516‐522. doi:10.1016/j.jhep.2020.03.049 32531415 PMC7438303

[72] Christians JK , Lennie KI , Wild LK , Garcha R . Effects of high‐fat diets on fetal growth in rodents: a systematic review. Reprod Biol Endocrinol. 2019;17(1):39. doi:10.1186/s12958-019-0482-y 30992002 PMC6469066

[73] Hay WW , Meznarich HK . Effect of maternal glucose concentration on uteroplacental glucose consumption and transfer in pregnant sheep. Proc Soc Exp Biol Med. 1989;190(1):63‐69. doi:10.3181/00379727-190-42830 2911609

[74] Sferruzzi‐Perri AN , Vaughan OR , Haro M , et al. An obesogenic diet during mouse pregnancy modifies maternal nutrient partitioning and the fetal growth trajectory. FASEB J. 2013;27(10):3928‐3937. doi:10.1096/fj.13-234823 23825226

[75] Aldoretta PW , Hay WWJ . Chronic hyperglycemia induces insulin resistance and glucose intolerance in fetal sheep. Pediatr Res. 2001;49(307A):1758.

[76] Kua KL , Hu S , Wang C , et al. Fetal hyperglycemia acutely induces persistent insulin resistance in skeletal muscle. J Endocrinol. 2019;242(1):M1‐M15. 30444716 10.1530/JOE-18-0455 PMC6494731

[77] Hay WW . Placental‐fetal glucose exchange and fetal glucose metabolism. Trans Am Clin Climatol Assoc. 2006;117:321‐340. 18528484 PMC1500912

[78] Catalano PM , Presley L , Minium J , de Mouzon SH . Fetuses of obese mothers develop insulin resistance in utero. Diabetes Care. 2009;32(6):1076‐1080. doi:10.2337/dc08-2077 19460915 PMC2681036

[79] Aykroyd BRL , Tunster SJ , Sferruzzi‐Perri AN . Igf2 deletion alters mouse placenta endocrine capacity in a sexually dimorphic manner. J Endocrinol. 2020;246(1):93‐108. doi:10.1530/JOE-20-0128 32380473

[80] Shafrir E , Barash V . Placental glycogen metabolism in diabetic pregnancy. Isr J Med Sci. 1991;27(8–9):449‐461. 1835720

[81] Akison LK , Nitert MD , Clifton VL , Moritz KM , Simmons DG . Review: Alterations in placental glycogen deposition in complicated pregnancies: current preclinical and clinical evidence. Placenta. 2017;54:52‐58. doi:10.1016/j.placenta.2017.01.114 28117144

[82] Pickard MR , Leonard AJ , Ogilvie LM , et al. Maternal hypothyroidism in the rat influences placental and liver glycogen stores: fetal growth retardation near term is unrelated to maternal and placental glucose metabolic compromise. J Endocrinol. 2003;176(2):247‐255. doi:10.1677/joe.0.1760247 12553873

[83] Tunster SJ , Watson ED , Fowden AL , Burton GJ . Placental glycogen stores and fetal growth: insights from genetic mouse models. Reproduction. 2020;159(6):R213‐R235. 32191912 10.1530/REP-20-0007

[84] Perez PA , DiPatrizio NV . Impact of maternal western diet‐induced obesity on offspring mortality and peripheral endocannabinoid system in mice. PLOS ONE. 2018;13(10):e0205021. 30273406 10.1371/journal.pone.0205021 PMC6166980

[85] DiPatrizio NV , Piomelli D . The thrifty lipids: endocannabinoids and the neural control of energy conservation. Trends Neurosci. 2012;35(7):403‐411. doi:10.1016/j.tins.2012.04.006 22622030 PMC3744874

[86] Jevitt C , Hernandez I , Groër M . Lactation complicated by overweight and obesity: supporting the mother and newborn. J Midwifery Womens Health. 2007;52(6):606‐613. doi:10.1016/j.jmwh.2007.04.006 17983998

[87] Macias H , Hinck L . Mammary gland development. Wiley interdisciplinary reviews. Dev Biol. 2012;1(4):533‐557. doi:10.1002/wdev.35 PMC3404495 22844349

[88] Hue‐Beauvais C , Chavatte‐Palmer P , Aujean E , et al. An obesogenic diet started before puberty leads to abnormal mammary gland development during pregnancy in the rabbit. Dev Dyn. 2011;240(2):347‐356. doi:10.1002/dvdy.22536 21246651

[89] Boumahrou N , Andrei S , Miranda G , et al. The major protein fraction of mouse milk revisited using proven proteomic tools. J Physiol Pharmacol. 2009;60(Suppl. 3):113‐118. 19996491

[90] Kolb AF , Huber RC , Lillico SG , et al. Milk lacking α‐casein leads to permanent reduction in body size in mice. PLoS ONE. 2011;6(7):e21775. 21789179 10.1371/journal.pone.0021775 PMC3138747

[91] Moazzam S , Jarmasz JS , Jin Y , Siddiqui TJ , Cattini PA . Effects of high fat diet‐induced obesity and pregnancy on prepartum and postpartum maternal mouse behavior. Psychoneuroendocrinology. 2021;126:105147. doi:10.1016/j.psyneuen.2021.105147 33497916

[92] Nilsson J , Ericsson M , Joibari MM , et al. A low‐carbohydrate high‐fat diet decreases lean mass and impairs cardiac function in pair‐fed female C57BL/6J mice. Nutr Metab. 2016;13(1):79. doi:10.1186/s12986-016-0132-8 PMC5111238 27891164

[93] Gonzalez PN , Gasperowicz M , Barbeito‐Andrés J , Klenin N , Cross JC , Hallgrímsson B . Chronic protein restriction in mice impacts placental function and maternal body weight before fetal growth. PLoS ONE. 2016;11(3):e0152227. doi:10.1371/journal.pone.0152227 27018791 PMC4809512

[94] Watkins AJ , Wilkins A , Cunningham C , et al. Low protein diet fed exclusively during mouse oocyte maturation leads to behavioural and cardiovascular abnormalities in offspring. J Physiol. 2008;586(8):2231‐2244. doi:10.1113/jphysiol.2007.149229 18308825 PMC2465188

[95] Watkins AJ , Lucas ES , Torrens C , et al. Maternal low‐protein diet during mouse pre‐implantation development induces vascular dysfunction and altered renin‐angiotensin‐system homeostasis in the offspring. Br J Nutr. 2010;103(12):1762‐1770. doi:10.1017/S0007114509993783 20128937

[96] Sasson IE , Vitins AP , Mainigi MA , Moley KH , Simmons RA . Pre‐gestational vs gestational exposure to maternal obesity differentially programs the offspring in mice. Diabetologia. 2015;58(3):615‐624. doi:10.1007/s00125-014-3466-7 25608625 PMC4452998

[97] Napso T , Lean SC , Lu M , et al. Diet‐induced maternal obesity impacts feto‐placental growth and induces sex‐specific alterations in placental morphology, mitochondrial bioenergetics, dynamics, lipid metabolism and oxidative stress in mice. Acta Physiol. 2022;234(4):e13795. doi:10.1111/apha.13795 PMC9286839 35114078

[98] Kay RG , Challis BG , Casey RT , et al. Peptidomic analysis of endogenous plasma peptides from patients with pancreatic neuroendocrine tumours. Rapid Commun Mass Spectrom. 2018;32(16):1414‐1424. 29857350 10.1002/rcm.8183 PMC6099210

[99] de Clercq K , Lopez‐Tello J , Vriens J , Sferruzzi‐Perri AN . Double‐label immunohistochemistry to assess labyrinth structure of the mouse placenta with stereology. Placenta. 2020;94:44‐47. doi:10.1016/j.placenta.2020.03.014 32421534

[100] Coan PM , Ferguson‐Smith AC , Burton GJ . Developmental dynamics of the definitive mouse placenta assessed by stereology. Biol Reprod. 2004;70(6):1806‐1813. doi:10.1095/biolreprod.103.024166 14973263

