# Supplementary material for: Obesogenic diet in mice compromises maternal metabolic physiology and lactation ability leading to reductions in neonatal viability
Source: Acta Physiol (Oxf). 2022 Aug 3;236(2):e13861. doi: 10.1111/apha.13861 (PMC9787084; doi:10.1111/apha.13861)
Supplement: Supplementary file 1 — Supinfo [file APHA-236-e13861-s003.docx]

**Supplementary Table 1:** Maternal body, adipose depot, and organ weights at E18.5

|  | **Control (n=10)** | **HFHS (n=10)** | **P** |
| --- | --- | --- | --- |
| **Body composition** | | | |
| Body Weight  Whole  Hysterectomised | 36.55 ± 0.81  23.18 ± 0.40 | 35.16 ± 0.93  22.67 ± 0.53 | NS  NS |
| **Adipose depot** | | | |
| Renal (mg)  % hyster | 80.0 ± 6.9  0.33 ± 0.03 | 81.7 ± 7.5  0.36 ± 0.03 | NS  NS |
| Retro (mg)  % hyster | 35.5 ± 3.9  0.14 ± 0.01 | 47.3 ± 4.9  0.22 ± 0.02 | **0.0320**  **0.0191** |
| Gonadal (mg)  % hyster | 160.6 ± 9.3  0.70 ± 0.04 | 177.6 ± 13.8  0.78 ± 0.05 | NS  NS |
| **Maternal organ** | | | |
| Brain (mg)  % hyster | 402.9 ± 8.08  1.74 ± 0.04 | 429.1 ± 7.97  1.90 ± 0.02 | **0.0333**  **0.0031** |
| Heart (mg)  % hyster | 113.6 ± 3.35  0.49 ± 0.01 | 133.5 ± 6.63  0.59 ± 0.03 | **0.0155**  **0.0064** |
| Liver (mg)  % hyster | 1828 ± 38.37  7.88 ± 0.10 | 2007 ± 87.24  8.86 ± 0.36 | NS  **0.0172** |
| Spleen (mg)  % hyster | 65.9 ± 3.22  0.28 ± 0.02 | 64.8 ± 4.32  0.29 ± 0.01 | NS  NS |
| Kidney (mg)  % hyster | 132.4 ± 1.89  0.29 ± 0.004 | 127.4± 4.02  0.28 ± 0.01 | NS  NS |
| Adrenals (mg)  % hyster | 4.77 ± 0.3  0.01 ± 0.001 | 4.86 ± 0.3  0.01 ± 0.001 | NS  NS |
| Muscle (mg)  % hyster | 137.3 ± 10.8  0.59 ± 0.04 | 127.8 ± 4.94  0.57 ± 0.03 | NS  NS |
| Pancreas (mg)  % hyster | 201.6 ± 9.23  0.87 ± 0.030 | 168.6 ± 10.31  0.75 ± 0.05 | **0.0287**  **0.0422** |
| Mammary (mg)  % hyster | 280.5 ± 14.77  0.61 ± 0.03 | 327.6 ± 23.72  0.72 ± 0.05 | NS  NS |

Retro= Retroperitoneal. Kidney, adrenal and (abdominal) mammary gland presented as mean of left and right organs. Muscle = right quadricep. NS = not significant. Data presented as % of hysterectomised (hyster) body weight, mean±SEM, analysed by students t-test, significance at p<0.05.

**Supplementary Table 2:** Specific reaction monitoring (SRM) information for the analysis of digested mammary gland extracts showing Q1 and Q3 *m/z* values and associated collision energy settings.

| **Q1** | **Q3** | **Collision energy** | **Protein** |
| --- | --- | --- | --- |
| 439.6 | 543.35 | 20 | Alpha-S1- Casein |
| 439.6 | 573.85 | 10 | Alpha-S1- Casein |
| 600.27 | 480.72 | 20 | Alpha-S1- Casein |
| 600.27 | 529.25 | 20 | Alpha-S1- Casein |
| 1029.51 | 583.39 | 25 | Kappa casein |
| 1029.51 | 1040.56 | 35 | Kappa casein |
| 1163.28 | 331.2 | 50 | Gamma casein |
| 1163.28 | 1006.52 | 35 | Gamma casein |
| 509.79 | 159.09 | 20 | Epsilon Casein |
| 509.79 | 804.44 | 15 | Epsilon Casein |
| 1459.63 | 857.47 | 35 | Beta casein |
| 1459.63 | 1053.6 | 40 | Beta casein |
| 558.29 | 243.13 | 30 | Whey acidic protein |
| 558.29 | 715.77 | 10 | Whey acidic protein |
| 789.49 | 375.2 | 25 | Whey acidic protein |
| 789.49 | 964.42 | 15 | Whey acidic protein |
| 803.37 | 449.22 | 25 | Alpha lactalbumin |
| 803.37 | 907.38 | 30 | Alpha lactalbumin |
| 980.41 | 509.2 | 30 | Alpha lactalbumin |
| 980.41 | 1508.63 | 30 | Alpha lactalbumin |
| 901.4 | 244.16 | 25 | Lactotransferrin |
| 901.4 | 584.3 | 25 | Lactotransferrin |
| 661.65 | 699.41 | 20 | Serum albumin |
| 661.65 | 756.44 | 20 | Serum albumin |

**Figure legends for supplementary figures**

**Supplementary Figure 1:** Schematic showing the study design, in particular the timeline of the experimental protocol. HFHS = high fat/high sugar customised diet.

**Supplementary Figure 2:** Graphical abstract and results summary.
