# Supplementary figures and images for: Obesogenic diet in mice compromises maternal metabolic physiology and lactation ability leading to reductions in neonatal viability
Source: Acta Physiol (Oxf). 2022 Aug 3;236(2):e13861. doi: 10.1111/apha.13861 (PMC9787084; doi:10.1111/apha.13861)

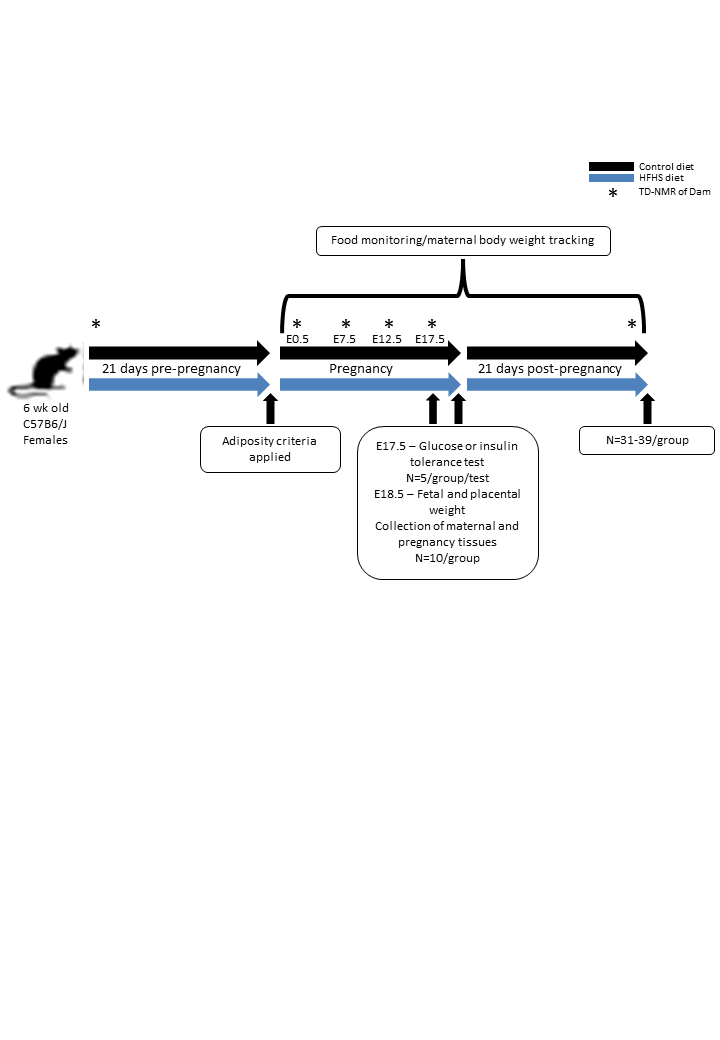

Supplement: Supplementary file 2 — Figure S1 [file APHA-236-e13861-s002.tif]

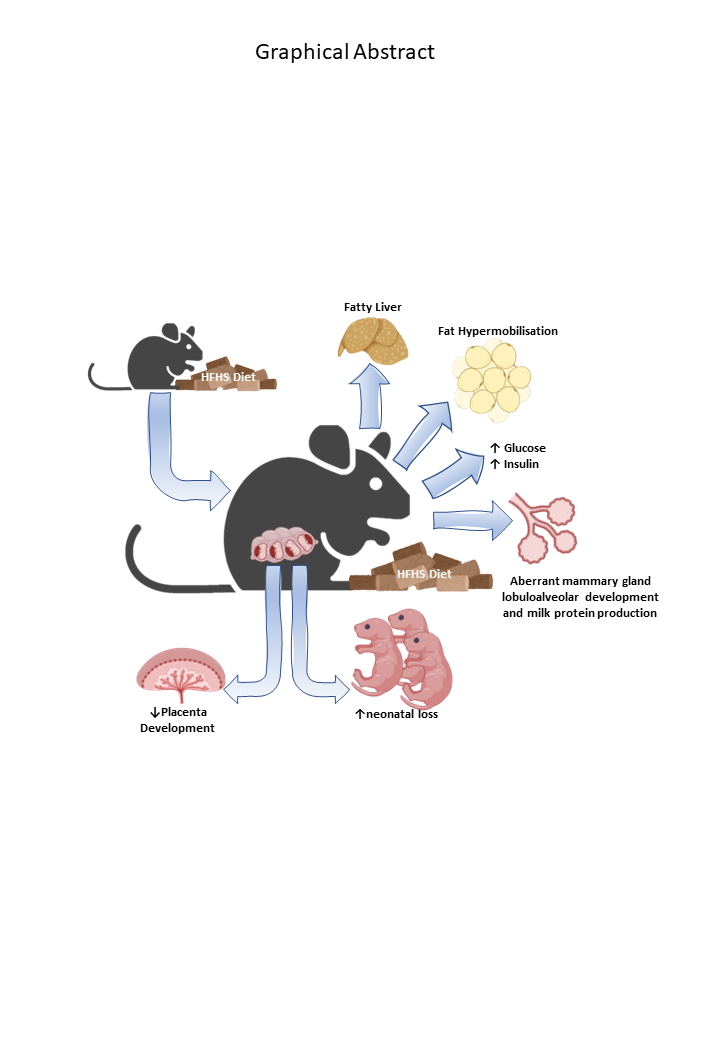

Supplement: Supplementary file 3 — Figure S2 [file APHA-236-e13861-s001.tif]
